# Genetic Rescue of Pathogenic O-GlcNAc Dyshomeostasis Associated with Microcephaly and Motor Deficits

**DOI:** 10.1523/ENEURO.0453-25.2026

**Published:** 2026-06-04

**Authors:** Florence Authier, Iria Esperon-Abril, Kévin-Sébastien Coquelin, Christian Stald Skoven, Simon Fristed Eskildsen, Nina Ondruskova, Andrew T. Ferenbach, Jesper Skovhus Thomsen, Brian Hansen, Daan M. F. van Aalten

**Affiliations:** ^1^Section for Neurobiology and DANDRITE, Department of Molecular Biology and Genetics, Aarhus University, 8000 Aarhus, Denmark; ^2^CFIN, Aarhus Hospital, 8200 Aarhus N, Denmark; ^3^Department of Paediatrics and Inherited Metabolic Disorders, First Faculty of Medicine, Charles University and General University Hospital in Prague, Prague, 128 08 Praha 2, Czech Republic; ^4^Department of Biomedicine, Aarhus University, 8000 Aarhus, Denmark; ^5^Division of Molecular, Cell and Developmental Biology, School of Life Sciences, University of Dundee, Dundee DD1 5EH, United Kingdom

**Keywords:** intellectual disability, mouse model, neurodevelopment, neuroscience, O-GlcNAc, OGT-CDG

## Abstract

Missense variants in O-GlcNAc transferase (OGT) result in OGT congenital disorder of glycosylation (OGT-CDG), an intellectual disability syndrome associated with O-GlcNAc dyshomeostasis and a range of neurodevelopmental defects. Inhibition of O-GlcNAcase (OGA), the enzyme responsible for removing protein O-GlcNAcylation, has been explored as a target for modulating brain O-GlcNAc homeostasis in neurodegenerative diseases and may also be a target for OGT-CDG. Here, we describe an OGT-CDG mouse line, studied in male mice, that exhibits microcephaly, motor deficits, and brain O-GlcNAc dyshomeostasis, closely mirroring patient symptoms. We genetically explored OGA as a target for OGT-CDG by crossing these mice with a line carrying catalytically inactive OGA. Encouragingly, this partially restored O-GlcNAc homeostasis in brain and blood as determined by *Ogt/Oga* mRNA ratio. These findings suggest that OGA inhibition can modulate enzymatic imbalance in OGT-CDG mice possessing microcephaly and motor deficits and that blood can be used to monitor the effects of interventions targeting O-GlcNAc dyshomeostasis.

## Significance Statement

O-GlcNAc is a chemical modification present on many proteins inside cells and is vital for healthy brain development. Mutations in the OGT enzyme, which adds this modification, lead to a neurodevelopmental disorder involving intellectual disability, microcephaly, and motor difficulties. Although >70 individuals with this condition are now known, we still lack a clear understanding of how OGT mutations cause the disease. As a result, there are currently no biomarkers or targeted treatments. Our research addresses these gaps by developing a disease-relevant mouse model and identifying measurable changes in O-GlcNAc homeostasis associated with brain function. This work provides crucial groundwork for future diagnosis and therapy.

## Introduction

The maintenance of cellular homeostasis is crucial for proper cell function and requires mechanisms that can respond rapidly to metabolic and environmental changes. This regulation is primarily controlled by posttranslational modifications (PTMs), which lead to rapid, reversible changes in protein dynamics. One such metabolism-responsive PTM is O-linked β-*N*-acetylglucosamine (O-GlcNAc; Torres and Hart, 1984; Hart et al., 1989). O-GlcNAc moieties are covalently attached to serine and threonine residues on thousands of proteins localized in the nucleus, cytoplasm, and mitochondria (Hart et al., 2007; Wulff-Fuentes et al., 2021). Unlike many other PTMs, O-GlcNAc cycling is controlled by only two enzymes: O-GlcNAc transferase (OGT), which adds the modification, and O-GlcNAcase (OGA), which removes it (Yang and Qian, 2017; Wu et al., 2024). O-GlcNAc integrates cellular nutrient availability and metabolic state, and its levels depend on the tight regulation of both OGT and OGA (Cheung and Hart, 2008; Taylor et al., 2009; Ruan et al., 2012; Hardivillé and Hart, 2014). Accordingly, protein O-GlcNAcylation is implicated in the regulation of numerous crucial cellular processes, including gene expression, stress responses, and metabolic pathways (Zachara and Hart, 2004; Ruan et al., 2012; Han et al., 2017) and is linked to a range of diseases (Nie and Yi, 2019; Wenzel and Olivier-Van Stichelen, 2022).

O-GlcNAcylation and its associated enzymes play a crucial role in normal development, as shown by the lethality of knock-out animal models (O’Donnell et al., 2004; Y. R. Yang et al., 2012; Keembiyehetty, 2015; Muha et al., 2021). The enzymes and the modification are particularly abundant in the brain (Cole and Hart, 2001; K. Liu et al., 2004; Vosseller et al., 2006), with several studies indicating that O-GlcNAc plays a role in neurodevelopment and synaptic function (Francisco et al., 2009; Liu et al., 2012, 2017; Lagerlöf and Hart, 2014; Savage et al., 2018). Accordingly, the levels of O-GlcNAc, OGT, and OGA are tightly regulated during mammalian brain development, showing a gradual decline of global O-GlcNAcylation with age (Liu et al., 2012). Disrupted O-GlcNAc levels have been associated with several neurodegenerative diseases, including Parkinson's and Alzheimer's disease (AD; Levine et al., 2019; Lee et al., 2021). AD is characterized, among other features, by reduced O-GlcNAc levels in the brain, which have been linked to increased necroptosis, neuroinflammation, and tau pathology (Park et al., 2021). O-GlcNAcylation of tau anti-correlates with pathogenic hyperphosphorylation, suggesting that the reduction in O-GlcNAc levels in AD could contribute to tauopathies (F. Liu et al., 2004; Park et al., 2021; Rostgaard et al., 2023). In mouse models of AD, increasing O-GlcNAc by chronic treatment with OGA inhibitors had a neuroprotective effect against both tau and amyloid-β peptide pathology (Yuzwa et al., 2012, 2014; Hastings et al., 2017; Park et al., 2021), although most of these inhibitors have now failed in the clinic due to neurotoxicity (Meade et al., 2025). Similar conclusions were drawn when examining the impact of increasing O-GlcNAc on Parkinson's disease pathology (Marotta et al., 2015; Lee et al., 2020; Tavassoly et al., 2021; Kim et al., 2024), epilepsy (Stewart et al., 2017; Sanchez et al., 2019), ALS (Shan et al., 2012; Hsieh et al., 2019; Hao et al., 2025), and Down syndrome (Zuliani et al., 2021; Lanzillotta et al., 2025). In contrast, other studies indicate that OGA inhibition is detrimental to neurons via α-synuclein accumulation (Wani et al., 2017) and that decreased O-GlcNAcylation has a protective effect in animal models of other neurodegenerative disorders (Wang et al., 2012; Kumar et al., 2014). Overall, the mechanisms mediating the impact of O-GlcNAc in neurodegenerative diseases are not well understood, as changes in O-GlcNAc homeostasis may have context- and disease-dependent impacts.

Recently, pathogenic missense variants in the *OGT* gene have been linked to a syndromic form of X-linked intellectual disability (ID), known as O-GlcNAc transferase congenital disorder of glycosylation (OGT-CDG; Pravata et al., 2020). The OGT-CDG syndrome is characterized by several neurological and psychiatric manifestations, such as developmental delay, autism spectrum disorder (ASD), and epilepsy, as well as locomotion defects and behavioral deficits. Several OGT-CDG variants have been clinically described (Vaidyanathan et al., 2017; Willems et al., 2017; Selvan et al., 2018; Pravata et al., 2019a,b; Authier et al., 2024), revealing that patients typically exhibit a range of additional facial and skeletal abnormalities, including microcephaly, craniofacial dysmorphia, clinodactyly, and growth impairment (Pravata et al., 2020; Mayfield et al., 2024). There are no treatments available for this group of patients, and it is not yet known whether the neurological symptoms are primarily of neurodevelopmental or neurophysiological origin. When modeled in stem cells, *Drosophila*, and mice, these missense variants cause O-GlcNAc dyshomeostasis, presumably as a result of reduced OGT activity, and it is assumed that loss of O-GlcNAc on key proteins contributes to the neurological phenotypes observed, through mechanisms that remain to be dissected (Pravata et al., 2019a; Fenckova et al., 2022; Omelková et al., 2023; Authier et al., 2024; Czajewski et al., 2024; Mayfield et al., 2024).

In OGT-CDG, several characterized variants result in a decrease in OGA protein levels (Pravata et al., 2019a,b; Authier et al., 2024), likely exploited as a compensatory mechanism by cells to attempt to restore O-GlcNAc homeostasis. This would suggest that inhibiting OGA in the context of OGT-CDG may be a potential method for (further) correcting O-GlcNAc imbalance. However, it is not clear whether such OGA inhibition would be required during early development to compensate for neurodevelopmental defects during embryogenesis and/or in adulthood to compensate for neurophysiological defects. We sought a genetic strategy to dissect these possible mechanisms and explore OGA as a target for OGT-CDG. We have previously reported an OGA catalytically deficient mouse (*Oga*^D285A^) generated through a constitutive knock-in of an Asp285Ala mutation at an active site residue that is key for both catalytic activity and O-GlcNAc binding (Muha et al., 2021). Homozygous *Oga*^D285A/D285A^ mice, like the previously reported *Oga* knock-out mice (Y. R. Yang et al., 2012), die perinatally, emphasizing the crucial role of OGA activity during development. However, heterozygous *Oga*^D285A/+^ mice develop normally and exhibit tissue-specific changes in O-GlcNAc homeostasis regulation to compensate for OGA catalytic deficiency (Muha et al., 2021). Here, we used these mice to restore O-GlcNAc homeostasis in a mouse model of OGT-CDG. To achieve this, we first generated a new OGT-CDG mouse line carrying the N648Y patient variant (*Ogt*^N648Y/y^). This de novo variant leads to the nonconservative substitution of Asn648 in the OGT active site with Tyr, resulting in a significant loss of activity of the enzyme, hypo-O-GlcNAcylation in a stem cell model, and significant neurodevelopmental deficits in the patient (Pravata et al., 2019a). *Ogt*^N648Y/y^ mice exhibit a reduction in brain, skull, and body size, brain and blood O-GlcNAc levels, and behavioral defects that recapitulate symptoms seen in the patient, such as hyperactivity and motor deficits. We generated *Ogt*^N648Y/y^*Oga*^D285A/+^ mice, lacking 50% of OGA activity from embryogenesis to adulthood, as a genetic approach to compensate for the hypo-O-GlcNAcylation seen in the *Ogt*^N648Y/y^ mice. Although this did not lead to improvements in OGT-CDG-related behavioral deficits or body weight, we found that this approach did partially restore O-GlcNAc homeostasis in the brain and blood, emphasizing the potential of blood samples as a disease biomarker and evaluating the efficiency of future treatments for OGT-CDG.

## Materials and Methods

### Generation of mouse lines and animal husbandry

*Ogt*^N648Y/y^ mice were produced by microinjection as described previously (Authier et al., 2024). Genomic DNA from offspring was genotyped and sequenced to confirm the presence of the OGT^N648Y^ variant. Editing CRISPR reagents, Cas9 Nickase (1081062; Integrated DNA Technologies), and primers were purchased from Integrated DNA Technologies. The sequences for the editing reagents are as follows: GAG CTC ATT CCG AGC ACC CT (guide RNA, left), TCT TAG GCC AGC TCC TAT TC (guide RNA, right), and CCA CAG ATT CCT TGT AAT GGA AAA GCA GCC GAC CGC ATC CAC CAA GAT GGA ATT CAC ATC CTT GTG AAT ATG AAT GGG TAT ACA AAA GGC GCC CGC TAT GAG CTC TTT GCT CTT AGG CCT GCC CCC ATC CAG GTA AAA GAA CAA TCA CTT ACA ATG TCT ATT GGT CTG AAA AGA TAG TGG GTT TTG GGT TTT CTC CAT C (DNA repair template for N648Y). Founder *Ogt*^N648Y/y^ mice were crossed to C57BL/6JRj WT animals (Janvier) for further breeding. *Oga*^D285A^ mice were previously reported (Muha et al., 2021) and were maintained under C57BL/6JRj background. Mice carrying the double mutation (*Ogt*^N648Y/y^*Oga*^D285A/+^) were generated by crossing *Ogt*^N648Y/+^ females with heterozygous *Oga*^D285A/+^ males, leading to *Ogt*^N648Y/y^*Oga*^D285A/+^ animals. Mice were housed in ventilated cages with water and food available ad libitum and 12 h light/dark cycles in the Skou Animal Facility (Aarhus University). All animal studies and breeding were performed following the ARRIVE guidelines and the European Communities Council Directive (2010/EU) and were approved by the Danish Animal Experiments Inspectorate (Dyreforsøgstilsynet), under breeding license 2022-15-0202-00135 and project license 2023-15-0201-01426.

### Tissue collection and disruption for protein and RNA analysis

Mice were killed by intraperitoneal injection of pentobarbital (overdose). Brain tissue from male mice was rapidly dissected, snap frozen in liquid nitrogen, and stored at −80°C. Tissues were dissociated in phosphate-buffered saline (PBS) using Precellys 24 Touch homogenizer (Bertin Technologies) as described previously (Authier et al., 2025). Brain homogenates were split in half for further protein and RNA extractions.

### Leukocyte extraction from peripheral blood

Whole blood was collected in heparinized tubes from intracardiac puncture. Then, 0.5 ml of blood was lysed in 1 ml of erythrocyte lysis buffer (ELB; in mM: 155 NH_4_Cl, 10 NaHCO_3_, 1 EDTA, pH 7.4). After a short mix, samples were incubated on ice for 10 min and spun at 1,500 × *g* for 5 min at 4°C. Pellets were resuspended in ELB, mixed, and spun at 1,500 × *g* for 5 min. Finally, pellets were resuspended in 350 µl lysis buffer from the RNeasy Mini kit (74104, Qiagen) and stored at −80°C until further RNA processing.

### Western blot

Brain homogenates were lysed using 10× RIPA buffer (Cell Signaling), sonicated (10s on/10 s off at 20% amplitude, 6 pulses), and centrifuged at 14,000 rpm for 30 min at 4°C. Proteins were quantified, separated, and transferred to nitrocellulose membranes as described previously (Authier et al., 2025). The antibodies used were as follows: anti-OGA (MGEA5; 1:500 dilution; HPA036141; Sigma), anti-O-GlcNAc (RL2; 1:500 dilution; NB300-524, Novus Biologicals), anti-OGT (F-12; 1:1,000 dilution; sc-74546; Santa Cruz Biotechnology), mouse anti-Lamin B (1:10,000 dilution; 66095-Ig; Proteintech), and rabbit anti-Lamin B (1:10,000; 12987-1-AP; Proteintech). Blots were imaged using a Li-Cor Odyssey infrared imaging system (LI-COR), and signals were quantified using Empiria Studio software from the same manufacturer. Data were normalized to the mean of each WT replicate group and expressed as fold change relative to WT.

### RT-qPCR analysis

Total RNA was purified from brain homogenates, followed by reverse transcription and quantitative PCR as described previously (Authier et al., 2024, 2025). Samples were analyzed in biological replicates with technical triplicates using the comparative Ct method. The threshold-crossing value was normalized to internal control transcripts (*18S*, *Actb*, and *Pgk1*). Data were normalized to the mean of each WT replicate group and expressed as fold change relative to WT.

### Behavioral studies

All animals were handled daily by the experimenter for a week before behavior testing. Handling occurred in the experimental room with ambient lighting of 25–30 lux. Animals were housed in groups of 2–3 individuals. Behavioral testing was conducted over 5 consecutive weeks, with the mice aged between 10 and 14 weeks. Animals were placed in the experimental room at least 30 min before testing began. Data analysis was performed blindly with regard to genotype. The sample size was determined based on previous experience and confirmed through Post-hoc Power Calculation using the ClinCalc online tool (https://clincalc.com/stats/Power.aspx). Behavior tests to assess locomotion and motor coordination (open field, rotarod, static rods), cognitive performance (novel object recognition), anxiety (elevated maze, dark/light test), and compulsive behavior (marbles, digging and self-grooming, nesting) were performed as described previously (Authier et al., 2025). Gait analysis was performed using a 40-cm-long, 5.5-cm-wide runway with a dark box at the end, covered with a strip of paper. The day before the test, the mice were trained to walk through the illuminated runway toward the dark box in a straight line. On the test day, the underside of the forepaws (red) and hindpaws (blue) were painted, allowing them to walk along the corridor afterward. Several measurements between the footprints were analyzed, including stride length, step width, rear paws, and front paws base of support (RBOS and FBOS, respectively) and step alternation.

### Brain perfusion and sample preparation for structural analysis

Following behavior studies, *Ogt*^N648Y/y^ mice (WT *n* = 12; *Ogt*^N648Y/y^ *n* = 12) were anesthetized and perfused fixed for magnetic resonance imaging (MRI) as described previously (Authier et al., 2025). Briefly, transcardial perfusion fixation was followed by decapitation, after which the mandible and extracranial tissue were removed from the skull to prevent imaging artifacts. Hereafter, the in-skull brains were stored in 4% paraformaldehyde solution for at least 1 week. Before imaging, the samples were washed in PBS for a minimum of 24 h to increase MRI signal by removing excess fixative (Shepherd et al., 2009).

### Magnetic resonance imaging and image analysis

MRI scans were acquired on a 9.4 T preclinical system (BioSpec 94/20, Bruker Biospin) with a bore-mounted 25 mm quadrature transmit-receive coil. Structural data were collected as described previously (Authier et al., 2025) and aligned using precise multi-atlas segmentation (Ma et al., 2014). For all scan types, data quality was confirmed through visual inspection, and samples were rescanned if necessary to ensure consistently high data quality for subsequent analyses. Volumetric analyses were performed as previously described (Authier et al., 2025) using an in-house pipeline. Briefly, high-resolution FLASH images were preprocessed with B1 inhomogeneity correction, denoising, intensity normalization, and spatial alignment to a C57BL/6J mouse template via linear and nonlinear registration. Neuroanatomical labels were then transformed back to native space to extract absolute and relative regional volumes (region size as % of total brain volume), accounting for differences in brain size. Cortical thickness was measured as previously described (Authier et al., 2025), generating statistical maps of group differences by fitting a general model at each surface vertex (SurfStat, http://www.math.mcgill.ca/keith/surfstat/). Due to multiple comparisons, statistical maps were familywise error (FWE) corrected using random field theory (Worsley et al., 1996) with α = 0.001 as the cluster-defining threshold. All statistical maps were thresholded at *p* = 0.05, both uncorrected and corrected.

### Micro computed tomography

Following MRI, the in-skull brain samples were imaged using micro computed tomography (microCT) (Scanco vivaCT 80) as described previously in detail (Authier et al., 2025). Skulls were imaged and analyzed in 3D Slicer (https://www.slicer.org), where skulls were isolated and exported as 3D models. Image processing was performed with the investigator blinded to the group distribution. For 3D visualization of differences between WT and *Ogt*^N648Y/y^ mice, models for the average shapes of WT and *Ogt*^N648Y/y^ mouse skulls were generated from the general Procrustes analysis (GPA) module. Skull shape and size differences were assessed both by computing the bone volume and by measuring distances between the 45 surface landmarks and performing Euclidean distance matrix analysis (EDMA). One WT skull was excluded from analysis due to accidental damage during sample preparation.

### Statistical analysis

Unless otherwise noted, statistical analyses were performed with Prism 9. D'Agostino and Pearson, Shapiro–Wilk, and Kolmogorov–Smirnov normality tests were conducted to verify normality. For data meeting normality requirements, the unpaired *t* test was used for pairwise comparisons of wild-type and mutant mouse data, and two-way ANOVA was used for multiple comparisons. For data sets that did not meet normality, the Mann–Whitney test was used for pairwise.

## Results

### *Ogt*^N648Y/y^ mice are viable and exhibit brain O-GlcNAc dyshomeostasis

**Figure 1.**
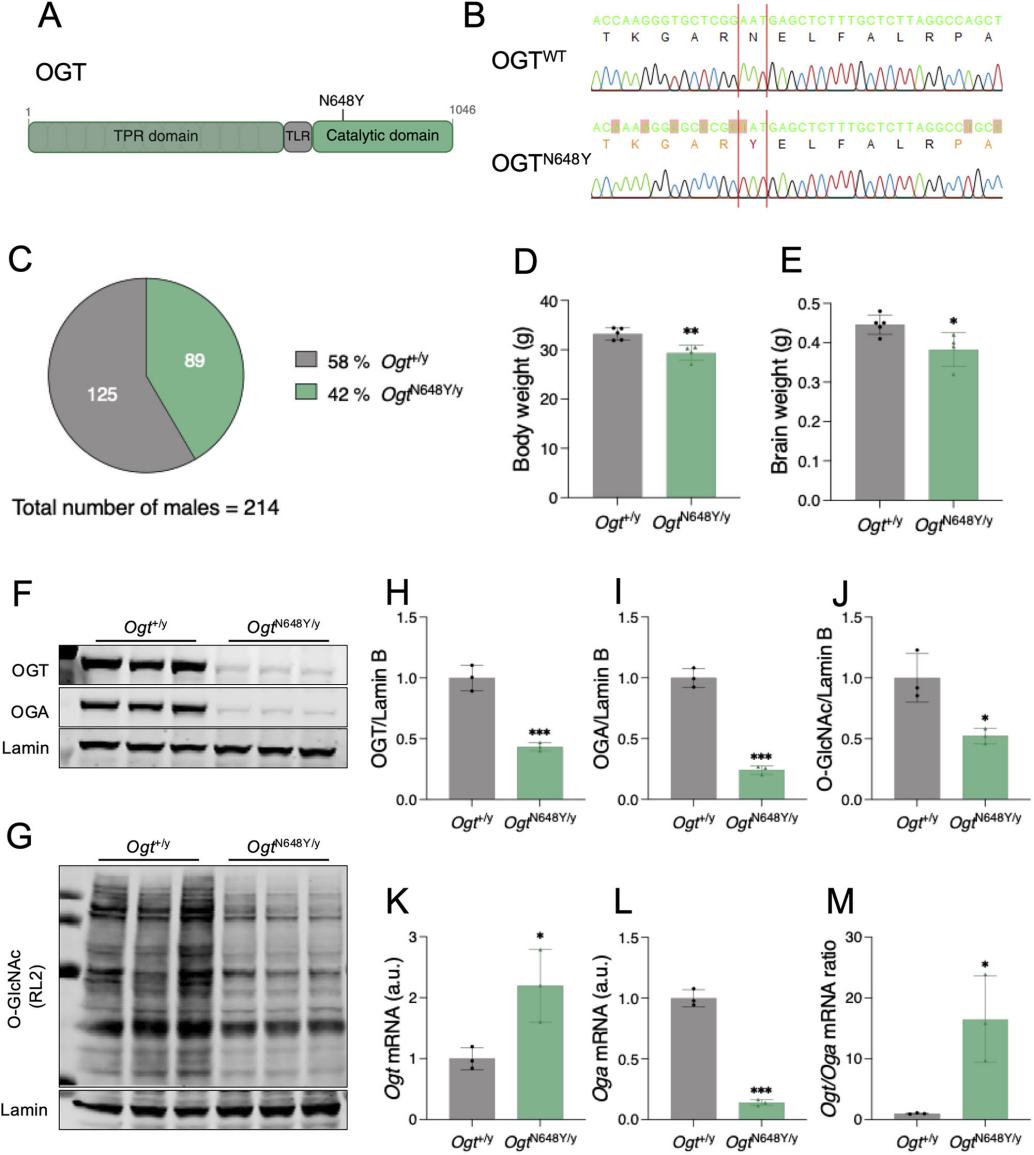
OGT^N648Y^ variant causes O-GlcNAc dyshomeostasis in the mouse brain. Data are represented as mean ± SD. Student’s *t* test is used for statistics. Significance is shown as follows: *p < 0.05, **p < 0.01, and ***p < 0.001. ***A***, Schematic of the OGT protein with the OGT^N648Y^ variant. The TPR (light green), TLR (gray), and catalytic domain (dark green) of OGT are represented. ***B***, Sequencing of genomic DNA of OGT^WT^ (top) and OGT^N648Y^ (bottom) confirms the presence of the N648Y point mutation (highlighted in red) in the transgenic animals. ***C***, Diagram showing numbers and percentages of *Ogt*^+/y^ and *Ogt*^N648Y/y^ animals generated from female *Ogt*^N648Y/+^ × male *Ogt*^+/y^ breeding pairs. ***D***, Measurement of body weight of *Ogt*^+/y^ (*n* = 5) and *Ogt*^N648Y/y^ (*n* = 4) mice. ***E***, Measurement of brain weight of *Ogt*^+/y^ (*n* = 5) and *Ogt*^N648Y/y^ (*n* = 4) mice. ***F***, Western blot of OGT and OGA levels in adult brain of *Ogt*^+/y^ (*n* = 3) and *Ogt*^N648Y/y^ (*n* = 3) mice. Lamin antibody was used as a loading control. ***G***, Western blot of O-GlcNAc levels in adult brain of *Ogt*^+/y^ (*n* = 3) and *Ogt*^N648Y/y^ (*n* = 3) mice. Lamin antibody was used as a loading control. ***H***, Quantification of OGT protein levels from the Western blot in panel ***F***. ***I***, Quantification of OGA protein levels from the Western blot in panel ***F***. ***J***, Quantification of O-GlcNAcylated proteins levels from the Western blot in panel ***G***. ***K***, Quantification of *Ogt* mRNA levels in whole mouse brain of *Ogt*^+/y^ (*n* = 3) and *Ogt*^N648Y/y^ (*n* = 3) by RT-PCR. ***L***, Quantification of *Oga* mRNA levels in whole mouse brain of *Ogt*^+/y^ (*n* = 3) and *Ogt*^N648Y/y^ (*n* = 3) by RT-PCR. ***M***, Quantification of *Ogt/Oga* mRNA ratio in whole mouse brain of *Ogt*^+/y^ (*n* = 3) and *Ogt*^N648Y/y^ (*n* = 3) by RT-PCR.

To investigate the effects of OGT-CDG variants in vivo, we focused on a recently described missense variant, N648Y, the first OGT-CDG variant reported within the catalytic domain of OGT (Pravata et al., 2019a). This variant was identified de novo in the second child of a nonconsanguineous family, where neither parent nor the older brother carried the variant and were all healthy. Facial asymmetry was evident shortly after birth, while other symptoms of the disease were noticeable only a few months later, as he showed a delay in achieving critical developmental milestones. In addition to physical features such as syndactyly and persistent drooling, the patient had no speech and presented moderate hyperactivity and increased sensitivity to external stimuli during early childhood. Given the severity of the clinical manifestations, the N648Y variant became a strong candidate for in vivo modeling. We used a CRISPR/Cas9 genome editing approach in mice to introduce this variant, generating cohorts of *Ogt*^N648Y/y^ males for phenotypic analyses (Fig. 1*A*). Briefly, pseudopregnant female mice were implanted with 0.5 d postcoitum zygotes injected with editing reagents. DNA from offspring was genotyped and sequenced to confirm the presence of the OGT^N648Y^ variant (Fig. 1*B*). As the gene encoding *OGT* is located on the X chromosome in both mouse and human, we evaluated the Mendelian inheritance distribution of male offspring generated from heterozygous *Ogt*^N648Y/+^ females crossed to wild-type (WT) *Ogt*^+/y^ males. We observed that the crosses generated 58% of male *Ogt*^+/y^ and 42% of male *Ogt*^N648Y/y^ animals, indicating a slightly reduced percentage of *Ogt*^N648Y/y^ animals born (Fig. 1*C*). We observed that *Ogt*^N648Y/y^ mice exhibit a decrease in body (Fig. 1*D*) and brain (Fig. 1*E*) weight compared with their WT littermates similar to that described in the recent first report of an OGT-CDG mouse model (Authier et al., 2024, 2025). Taken together, these results indicate that mice carrying the OGT^N648Y^ variant are viable but show reduced growth. Given the X-linked inheritance of *Ogt*, only WT and hemizygous *Ogt*^N648Y/y^ male animals were used for phenotypic analyses.

The OGT^N648Y^ variant was shown to affect OGT catalytic activity, leading to a reduction in OGA levels and hypoglycosylation in stem cells (Pravata et al., 2019a). Therefore, we assessed the levels of OGT, OGA, and O-GlcNAcylation in whole brains from adult WT and *Ogt*^N648Y/y^ animals. We observed that both OGT (Fig. 1*F*,*H*) and OGA (Fig. 1*F*,*I*) protein levels were significantly reduced in *Ogt*^N648Y/y^ mice compared with WT littermates. Likewise, global O-GlcNAcylated proteins were also significantly decreased in the *Ogt*^N648Y/y^ mice compared with WT (Fig. 1*G*,*J*). To determine whether the differences in OGT and OGA protein levels were due to impaired transcription, we performed qPCR analysis on whole-brain extracts. We found a significant increase in *Ogt* mRNA expression in *Ogt*^N648Y/y^ mouse brains (Fig. 1*K*), while *Oga* mRNA expression was decreased (Fig. 1*L*) compared with WT animals, leading to an increase in *Ogt/Oga* mRNA in *Ogt*^N648Y/y^ mice compared with WT—a sensitive measurement of O-GlcNAc dyshomeostasis (Fig. 1*M*). This indicates that the lower OGT protein levels seen in the *Ogt*^N648Y/y^ mutant are not caused by decreased *Ogt* mRNA expression but instead result from a posttranslational process. Taken together, these data suggest that while *Ogt*^N648Y/y^ mice are viable, they exhibit brain O-GlcNAc dyshomeostasis.

### *Ogt*^N648Y/y^ mice exhibit microcephaly associated with cortical hypoplasia

**Figure 2.**
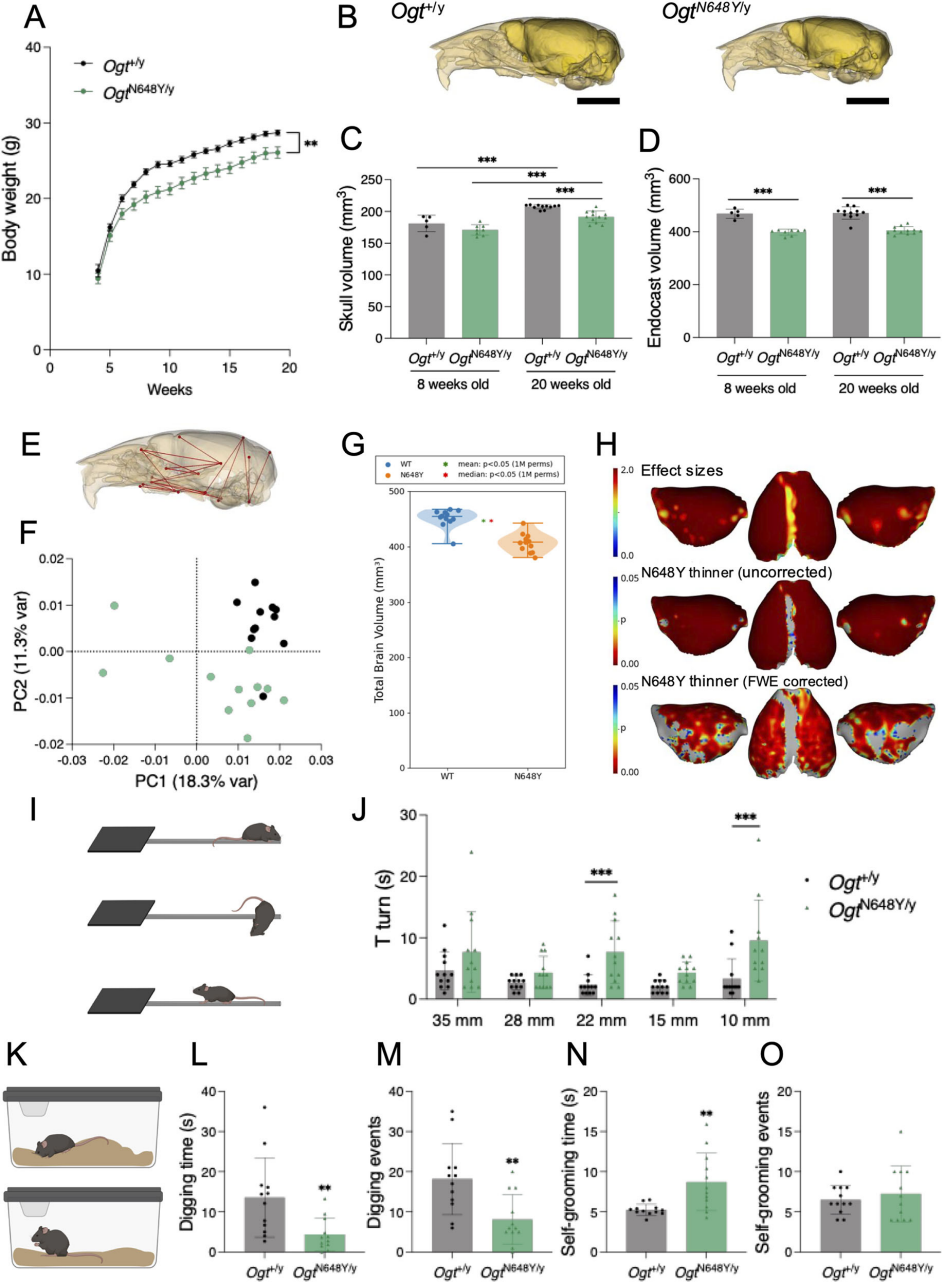
OGT^N648Y^ variant leads to microcephaly and motor deficits in mice. Data are represented as mean ± SD. The *n*-numbers are *n* = 5 and *n* = 8 for the 8-week-old *Ogt*^+/y^ and *Ogt*^N648Y/y^, respectively, and *n* = 12 for both genotypes at 20 weeks old. One *Ogt*^+/y^ skull was excluded from microCT analysis due to damage during sample processing. Student’s *t* test is used for statistics unless another test is indicated. Significance shown as follows: *p < 0.05, **p < 0.01, and ***p < 0.001. ***A***, Measurement of body weight over time of 12 *Ogt*^+/y^ and 12 *Ogt*^N648Y/y^ mice. Two-way ANOVA (alpha, 0.05) is used for statistics. ***B***, Lateral views of representative 3D reconstructions of the skull with endocast of *Ogt*^+/y^ (left) and *Ogt*^N648Y/y^ (right) mice. Scale bar: 5 mm. ***C***, Volume of the skulls of 8- and 20-week-old *Ogt*^+/y^ and *Ogt*^N648Y/y^ mice. ***D***, Volume of the endocasts of 8- and 20-week-old *Ogt* ^+^ ^y^ and *Ogt*^N648Y/y^ mice. ***E***, Lateral view of skull indicating linear distances at least 5% shorter in *Ogt*^N648Y/y^ mice compared with *Ogt*^+/y^. ***F***, PCA biplot of PC1 and PC2 of the surface landmarks of 20-week-old *Ogt*^+/y^ (black) and *Ogt*^N648Y/y^ (light green) mice. Percentages in the axis indicate the explained variance by the corresponding PC. ***G***, Total brain volume obtained by MRI. Statistical significance from permutation tests is indicated by asterisks (*, green for group mean, red for median). Data for absolute and relative volumetric measurements by brain regions are shown in Extended Data Figures 2-1–2-4. ***H***, Statistical maps of group differences in cortical thickness between *Ogt*^+/y^ and *Ogt*^N648Y/y^. Maps are effect sizes (top row), and *p* values of significant differences, both uncorrected for multiple comparisons (middle row), and with FWE correction. ***I***, Pictogram illustrating the static rods test. Mice are placed at the end of the rod (top), and the time required to perform the T-turn (middle) and reach the platform (bottom) is recorded. Created with BioRender. ***J***, Time to perform the T-turn of *Ogt*^+/y^ and *Ogt*^N648Y/y^ during the static rod tests. ***K***, Pictogram illustrating the digging and self-grooming tests. Created with BioRender. ***L***, Time spent digging by *Ogt*^+/y^ and *Ogt*^N648Y/y^ during 3 min observation. ***M***, Number of digging events by *Ogt*^+/y^ and *Ogt*^N648Y/y^ during 3 min observation. ***N***, Time spent self-grooming by *Ogt*^+/y^ and *Ogt*^N648Y/y^ during 3 min observation. ***O***, Number of self-grooming events by *Ogt*^+/y^ and *Ogt*^N648Y/y^ during 3 min observation. Data for other behavioral tests performed with these mice are detailed in Extended Data Figure 2-5.

To assess signs of global developmental delay, body weight was monitored weekly (Fig. 2*A*). In the first 2 weeks after weaning (4 to 5 weeks old), *Ogt*^N648Y/y^ and WT mice showed comparable body weights, whereas after 5 weeks old, lower gains in body weights in *Ogt*^N648Y/y^ mice were observed compared with their WT littermates (Fig. 2*A*), suggesting that *Ogt*^N648Y/y^ mice show general delayed postnatal development. OGT-CDG patients exhibit craniofacial abnormalities, including microcephaly, also seen in the patient carrying the OGT^N648Y^ variant (Pravata et al., 2019a). Therefore, we performed a comprehensive analysis of skull and brain morphometrics. We assessed skull shape and size using microCT imaging in *Ogt*^N648Y/y^ and WT mice at 8 weeks (WT, *n* = 5; *Ogt*^N648Y/y^, *n* = 8) and 20 weeks of age (WT, *n* = 11; *Ogt*^N648Y/y^, *n* = 12). For each animal, models were generated of the skull and the extracted endocranial cavity (endocast) as a proxy for brain volume (Fig. 2*B*). Measurement of the skull volume indicated that 20-week-old *Ogt*^N648Y/y^ mice show a significantly lower bone volume compared with their WT littermates. Similarly, 8-week-old *Ogt*^N648Y/y^ mice showed lower skull volume compared with WT animals, albeit not significant (Fig. 2*C*). Next, we measured the volume of the endocast as a proxy for brain volume. *Ogt*^N648Y/y^ mice exhibited 15% smaller endocasts than their WT littermates at both ages (Fig. 2*D*). Interestingly, the skull (bone) volume of mice from each genotype increased between 8 and 20 weeks of age, while the endocast volume remained unchanged (Fig. 2*C*,*D*), indicating that the bone continued to grow independently of the brain. To investigate whether the lower skull volume in the *Ogt*^N648Y/y^ mice was due to global or regional differences in size, we performed EDMA between 45 skull surface markers (Authier et al., 2025). At 20 weeks of age, 56% of the surface distances in *Ogt*^N648Y/y^ mouse skulls were at least 2% shorter compared with WT, with reductions of at least 5% observed in both the anteroposterior and vertical (cranial height) dimensions (Fig. 2*E*). To identify differences in skull shape independently of size differences, we used surface landmarks on the skulls to perform principal component analysis (PCA) in the 20-week-old mouse skulls (Fig. 2*F*). We observed that, while most of the WT mouse skulls separated along the first and second principal components (PC1 and PC2, respectively), *Ogt*^N648Y/y^ mouse skulls appeared more dispersed, indicating a higher morphological heterogeneity.

10.1523/ENEURO.0453-25.2026.f2-1 Figure 2-1 **Absolute volumetry of 39 brain regions from T1**. Each panel corresponds to a different bilaterally pooled region. Statistical significance (*p* < 0.05) from permutation tests (100k for each region) is indicated by asterisks (*; green for group mean, red for median) for uncorrected p-values. Correction for multiple comparisons was performed by division by the number of regions and is indicated by a pound symbol (#) or adjusted for false discovery rate (Benjamini-Hochsberg, BHcorr), indicated by a section sign (§) - if corrected, *p* < 0.05. Download Figure 2-1, TIF file.

10.1523/ENEURO.0453-25.2026.f2-2 Figure 2-2 **Table indicating absolute volumetry of 39 brain regions from T1** Mean and standard deviation of absolute volumes of the reported brain regions in *Ogt*^+/y^ and *Ogt*^N684Y/y^ mice, and significance of p-values after multiple comparisons correction (alpha, 0.05). Download Figure 2-2, DOCX file.

**Figure 3.**
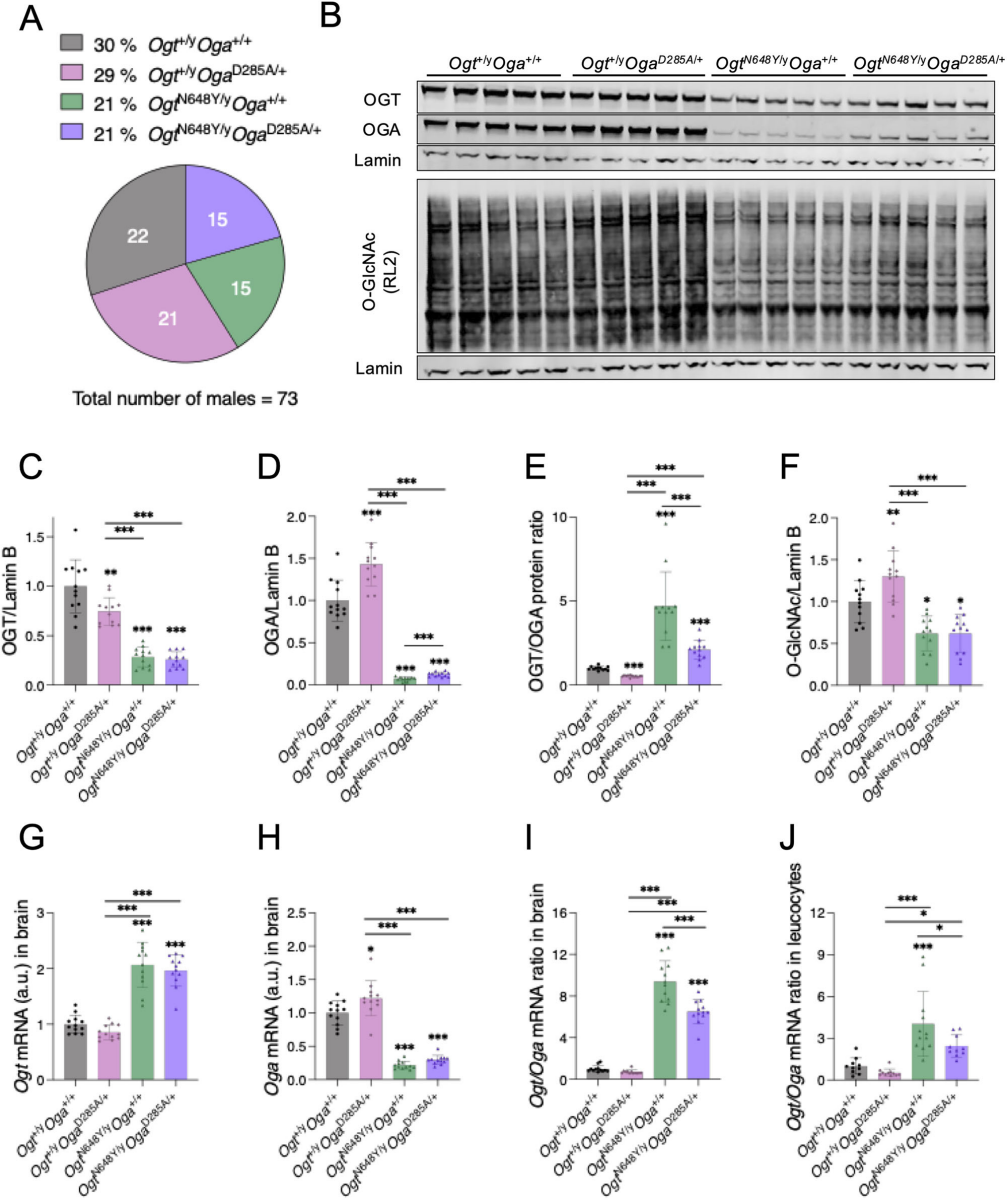
Genetic rescue of O-GlcNAc dyshomeostasis by crossing *Ogt*^N648Y/y^ mice with OGA-deficient mice. Data are represented as mean ± SD. The *n*-number is *n* = 12 for all genotypes. One-way ANOVA (alpha, 0.05) is used for statistics. Significance shown as follows: *p < 0.05, **p < 0.01, and ***p < 0.001. ***A***, Diagram indicating percentages and numbers of male *Ogt*^+/y^*Oga*^+/+^, *Ogt*^+/y^*Oga*^D285A/+^, *Ogt*^N648Y/y^*Oga*^+/+^, and *Ogt*^N648Y/y^*Oga*^D285A/+^ animals generated from female *Ogt*^N648Y/+^*Oga*^+/+^ × male *Ogt*^+/y^*Oga*^D285A/+^ breeding pairs. Data for these mice morphometrics are detailed in Extended Data Fig. 3-1. Data for the behavioral tests performed with these mice are detailed in Extended Data Fig. 3-2. ***B***, Western blot of OGT, OGA, and O-GlcNAcylated protein levels in adult brain of *Ogt*^+/y^*Oga*^+/+^, *Ogt*^+/y^*Oga*^D285A/+^, *Ogt*^N648Y/y^*Oga*^+/+^, and *Ogt*^N648Y/y^*Oga*^D285A/+^ mice. Lamin antibody was used as a loading control. ***C***, Quantification of OGT protein levels from the Western blot in panel ***B***. ***D***, Quantification of OGA protein levels from the Western blot in panel ***B***. ***E***, Quantification of OGT/OGA protein ratio from the Western blot in panel ***B***. ***F***, Quantification of O-GlcNAcylated proteins levels from the Western blot in panel ***B***. ***G***, Quantification of *Ogt* mRNA in whole mouse brain of *Ogt*^+/y^*Oga*^+/+^, *Ogt*^+/y^*Oga*^D285A/+^, *Ogt*^N648Y/y^*Oga*^+/+^, and *Ogt*^N648Y/y^*Oga*^D285A/+^ mice by RT-PCR. ***H***, Quantification of *Oga* mRNA in whole mouse brain of *Ogt*^+/y^*Oga*^+/+^, *Ogt*^+/y^*Oga*^D285A/+^, *Ogt*^N648Y/y^*Oga*^+/+^, and *Ogt*^N648Y/y^*Oga*^D285A/+^ mice by RT-PCR. ***I***, Quantification of *Ogt/Oga* mRNA ratio in whole mouse brain of *Ogt*^+/y^*Oga*^+/+^, *Ogt*^+/y^*Oga*^D285A/+^, *Ogt*^N648Y/y^*Oga*^+/+^, and *Ogt*^N648Y/y^*Oga*^D285A/+^ mice by RT-PCR. ***J***, Quantification of *Ogt/Oga* mRNA ratio in leukocytes of *Ogt*^+/y^*Oga*^+/+^, *Ogt*^+/y^*Oga*^D285A/+^, *Ogt*^N648Y/y^*Oga*^+/+^, and *Ogt*^N648Y/y^*Oga*^D285A/+^ mice by RT-PCR. Data for *Ogt* and *Oga* mRNA expression in blood is indicated in Extended Data Fig. 3-3.

10.1523/ENEURO.0453-25.2026.f2-3 Figure 2-3 **Relative volumetry of 39 brain regions from T1.** Each panel corresponds to a different bilaterally pooled region. The regional relative volume (RRV) is normalized to the individual total brain volume. Each dot corresponds to a subject. Statistical significance (*p* < 0.05) from permutation tests (100k for each region) is indicated by asterisks (*; green for group mean, red for median) for uncorrected p-values. Correction for multiple comparisons was performed by division by the number of regions and is indicated by a pound symbol (#) or adjusted for false discovery rate (Benjamini-Hochsberg, BHcorr) indicated by a section sign (§) - if corrected *p* < 0.05. Download Figure 2-3, TIF file.

10.1523/ENEURO.0453-25.2026.f2-4 Figure 2-4 **Table indicating relative volumetry of 39 brain regions from T1** Mean and standard deviation of relative volumes of the reported brain regions in *Ogt*^+/y^ and *Ogt*^N684Y/y^ mice, and significance of p-values after multiple comparisons correction (alpha, 0.05). Download Figure 2-4, DOCX file.

10.1523/ENEURO.0453-25.2026.f2-5 Figure 2-5 **Behaviour of *Ogt*^N648Y/y^ mice.** Data are represented as mean ± SD, n = 12 for all genotypes. Student’s t-test is used for statistics unless another test is indicated. Significance shown as: *p < 0.05, **p < 0.01 and ***p < 0.001 **(a)** Representative tracking plot of one *Ogt*^+/y^ and one *Ogt*^N648Y/y^ mouse over three days in the open field arena **(b)** Total distance travelled over three consecutive days in the open field arena of *Ogt*^+/y^ (black) and *Ogt*^N648Y/y^ (green) mice. Two-way ANOVA (alpha, 0.05) was used for statistics. Differences within *Ogt*^+/y^ mice over the days are indicated by a section sign (§), while differences among the *Ogt*^N648Y/y^ are indicated by a pound symbol (#). **(c)** Maximum speed achieved over three consecutive days in the open field arena by *Ogt*^+/y^ (black) and *Ogt*^N648Y/y^ (green) mice. Two-way ANOVA (alpha, 0.05) was used for statistics. Differences within *Ogt*^+/y^ mice over the days are indicated by a section sign (§), while differences among the *Ogt*^N648Y/y^ are indicated by a pound symbol (#). **(d)** Total time to perform the static rods tests of *Ogt*^+/y^ (black) and *Ogt*^N648Y/y^ (green) mice. **(e)** Delta time (total time – time to perform the T-turn) of *Ogt*^+/y^ (black) and *Ogt*^N648Y/y^ (green) mice during the static rods test. **(f)** Representative tracking plot of one *Ogt*^+/y^ and one *Ogt*^N648Y/y^ mouse during the elevated plus maze (EPM) test. **(g)** Total distance travelled of *Ogt*^+/y^ and *Ogt*^N648Y/y^ mice during the EPM test. **(h)** Number of entries to the centre of *Og*^t+/y^ and *Ogt*^N648Y/y^ mice during the EPM test. **(i)** Total time spent in open and closed arms of *Ogt*^+/y^ (black) and *Ogt*^N648Y/y^ (green) mice during the EPM test. **(j)** Representative tracking plot in the light compartment of one *Ogt*^+/y^ and one *Ogt*^N648Y/y^ mouse during the dark/light paradigm. **(k)** Distance travelled in the light of *Ogt*^+/y^ (black) and *Ogt*^N648Y/y^ (green) mice during the dark/light test. **(l)** Number of entries in the light of *Ogt*^+/y^ (black) and *Ogt*^N648Y/y^ (green) mice during the dark/light paradigm. **(m)** Time spent in the light of *Ogt*^+/y^ (black) and *Ogt*^N648Y/y^ (green) mice during the dark/light paradigm. **(n)** Number of buried marbles by *Ogt*^+/y^ and *Ogt*^N648Y/y^ mice during the marbles test. **(o)** Percentage of material removed by *Ogt*^+/y^ and *Ogt*^N648Y/y^ mice during the nesting test. **(p)** Percentage of litter removed by *Ogt*^+/y^ and *Ogt*^N648Y/y^ mice during the burrowing tube test. **(q)** Discrimination index of *Ogt*^+/y^ and *Ogt*^N648Y/y^ mice during the short-term (90 min) novel object (NOR) test. **(r)** Discrimination index of *Ogt*^+/y^ and *Ogt*^N648Y/y^ mice during the long-term (24 h) NOR test. Download Figure 2-5, TIF file.

Next, we turned to MRI to determine whether the reduction in brain size in the 20-week-old *Ogt*^N648Y/y^ mice is global or localized to specific brain regions. As expected, the *Ogt*^N648Y/y^ mice were found to have significantly smaller total brain volumes compared with WT littermates (Fig. 2*G*). Regional volumetric analysis showed a reduction in absolute volumes in most brain regions (Extended Data Fig. 2-1, 2-2). Regional relative volumes (normalized to total brain volume) in 39 bilaterally pooled brain regions were also determined (Extended Data Fig. 2-3, 2-4). The mean and median relative volumes were significantly different between the groups in the following brain regions: anterior commissure, cerebellar peduncle, inferior colliculus, fimbria, lateral septum, and olfactory bulbs. Considering only the means, differences were found in the corpus callosum and olfactory tubercle; while when only considering the medians, the areas that were significantly different were the midbrain and the periaqueductal gray. However, none of the differences in the regional relative volumes was significant when correcting for multiple comparisons (penalizing for the number of areas and Benjamini–Hochberg; Extended Data Fig. 2-4). Cortical thickness analysis revealed significantly thinner cortex over both hemispheres in the *Ogt*^N648Y/y^ mice compared with WT, with this difference remaining significant even after correcting for FWE (Fig. 2*H*). Taken together, these results suggest that the *Ogt*^N648Y/y^ mice exhibit a reduced skull size and shape deformations compared with their WT littermates, suggesting microcephaly and changes in skull shape, associated with cortical hypoplasia.

### *Ogt*^N648Y/y^ mice exhibit sensory motor deficits

Patients with OGT-CDG variants experience cognitive and motor impairments (Pravata et al., 2020). Therefore, we performed extensive behavioral screening of the *Ogt*^N648Y/y^ mice, including locomotion, memory, anxiety, and compulsive assessments to establish quantifiable phenotypic differences with their WT littermates. We first assessed general locomotion activity in an open field arena, where mice are placed in an open arena for 10 min for 3 consecutive days to evaluate exploratory drive in both novel and familiar environments. We observed an increase in the distance traveled and the maximum speed over the 3 d in *Ogt*^N648Y/y^ mice compared with their WT littermates (Extended Data Fig. 2-5*A–C*).

We next assessed sensory motor and coordination skills in the *Ogt*^N648Y/y^ mice using static rod tests, where mice are placed at the end of horizontal rods of decreasing diameters and are required to perform a T-turn before reaching a safety platform (Fig. 2*I*). We observed that although all *Ogt*^N648Y/y^ mice were able to perform the test, they required more time compared with WT mice as seen by an increase in total time for the 28, 22, 15, and 10 mm diameter rods (Extended Data Fig. 2-5*D*). This difference was driven by an increase in time to perform the motorically challenging T-turn in the *Ogt*^N648Y/y^ mice compared with WT (Fig. 2*J*), while the subsequent time to reach the safety platform was similar for both genotypes (Extended Data Fig. 2-5*E*).

In addition, we assessed anxiety-like behavior in the *Ogt*^N648Y/y^ mice using the elevated plus maze (EPM) test (Authier et al., 2025). We observed that *Ogt*^N648Y/y^ mice showed similar anxiety levels compared with WT mice, as seen by similar time spent in open and closed arms (Extended Data Fig. 2-5*F–I*), and comparable time spent in the light compartment in the dark/light paradigm (Extended Data Fig. 2-5*J–M*) between both genotypes. Of note, *Ogt*^N648Y/y^ traveled a greater distance and displayed more entries during the initial 5 min period of the dark/light test compared with WT. Similarly, *Ogt*^N648Y/y^ showed a mild increase in distance traveled and center entries in the EPM test, albeit not significant.

We then assessed repetitive behavior in the *Ogt*^N648Y/y^ mice. During a 3 min observation period (Fig. 2*K*), we observed a decrease both in the time spent digging (Fig. 2*L*) and in the number of digging events (Fig. 2*M*) in the *Ogt*^N648Y/y^ mice compared with WT littermates when mice were placed in a cage with 5 cm bedding to promote digging behavior. In addition, we observed an increase in the time spent self-grooming in the *Ogt*^N648Y/y^ mice (Fig. 2*N*), while the number of self-grooming events did not differ from those of the WT (Fig. 2*O*) when mice were placed in a cage with 1 cm bedding. We did not see any difference between *Ogt*^N648Y/y^ and WT mice in the number of buried marbles (Extended Data Fig. 2-5*N*). Finally, during both the nesting and burrowing tube tests, *Ogt*^N648Y/y^ mice removed the same amount of material as WT (Extended Data Fig. 2-5*O,P*).

Finally, we evaluated cognitive function in the mice by assessing novelty-associated memory using the novel object recognition (NOR) test. We did not observe a significant difference in the discrimination indexes for both short- and long-term paradigms (Extended Data Fig. 2-5*Q,R*), suggesting intact novelty-associated memory in the *Ogt*^N648Y/y^ mice. Taken together, these observations suggest that *Ogt*^N648Y/y^ mice show an impairment in fine sensory motor skills, accompanied by altered exploratory behavior and a degree of increased activity.

### Genetic rescue of O-GlcNAc dyshomeostasis in *Ogt*^N648Y/y^ mice

Both *Ogt*^N648Y/y^, described here, and *Ogt*^C921Y/y^ (Authier et al., 2025) OGT-CDG mouse models caused impaired brain O-GlcNAc dyshomeostasis. Lower O-GlcNAc levels and altered OGA/OGT ratios were observed, which could underpin the morphological and behavior deficits observed in the mouse models, although it is not yet clear whether these deficits are of neurodevelopmental or neurophysiological origin. Targeting OGA activity using small molecules has been widely used in neurodegenerative diseases to raise O-GlcNAc levels and improve cognitive function in models of neurodegeneration (Yuzwa et al., 2014; Hastings et al., 2017; Tavassoly et al., 2021). In an attempt to genetically validate this approach and rescue pathogenic O-GlcNAc dyshomeostasis in the *Ogt*^N648Y/y^ mice, we made use of the previously generated *Oga*^D285A^ mouse model (Muha et al., 2021). In the *Oga*^D285A^ mice, the key Asp285 residue that is required for both catalytic activity and O-GlcNAc binding has been replaced with Ala, leading to abolition of O-GlcNAcase activity and increased O-GlcNAc levels. Therefore, these mice can be exploited in a genetic strategy to model chronic inhibition of OGA activity from conception to adulthood. As homozygous *Oga*^D285A/D285A^ exhibit perinatal lethality, we used heterozygous *Oga*^D285A/+^ mice for subsequent breeding. Firstly, we assessed the viability and Mendelian inheritance distribution of male offspring generated from heterozygous *Ogt*^N648Y/+^ females crossed with heterozygous *Oga*^D285A/+^ males. DNA from the males in the offspring was genotyped and sequenced. Hemizygous *Ogt*^N648Y/y^ males, heterozygous *Oga*^D285A/+^ males, and the compound heterozygous *Ogt*^N648Y/y^*Oga*^D285A/+^ males could be obtained. We then analyzed Mendelian inheritance of WT *Ogt*^+/y^*Oga*^+/+^, heterozygous *Ogt*^+/y^*Oga*^D285A/+^, hemizygous *Ogt*^N648Y/y^*Oga*^+/+^, and compound heterozygous *Ogt*^N648Y/y^*Oga*^D285A/+^ male animals obtained from the crosses. Compared with the expected 25% per genotype, we observed that the crosses generated 30% *Ogt*^+/y^*Oga*^+/+^, 29% *Ogt*^+/y^*Oga*^D285A/+^, 21% *Ogt*^N648Y/y^*Oga*^+/+^, and 21% *Ogt*^N648Y/y^*Oga*^D285A/+^ male pups (chi-square = 2.3, *n* = 73; Fig. 3*A*), suggesting absence of lethality of the double *Ogt*^N648Y/y^*Oga*^D285A/+^ mutant animals and inheritance patterns similar to the *Ogt*^N648Y/y^ alone (Fig. 1*C*).

10.1523/ENEURO.0453-25.2026.f3-1 Figure 3-1 ***Ogt*^N648Y/y^*Oga*^D285A/+^** **mice** **morphometrics** Data for 20-week-old *Ogt*^+/y^*Oga*^+/+^, *Ogt*^+/y^*Oga*^D285A/+^, *Ogt*^N648Y/y^*Oga*^+/+^ and *Ogt*^N648Y/y^*Oga*^D285A/+^ mice unless otherwise stated. Data are represented as mean ± SD, n = 12 for all genotypes. One-way ANOVA (alpha, 0.05) is used for statistics unless another test is indicated. Significance shown as: *p < 0.05, **p < 0.01 and ***p < 0.001. **(a)** Measurement of body weight over time. Two-way ANOVA (alpha, 0.05) is used for statistics. **(b)** Measurement of body length at 20 weeks. **(c)** Measurement of skull length at 20 weeks. **(d)** Measurement of skull width at 20 weeks. **(e)** Measurement of body weight at 20 weeks **(f)** Measurement of brain weight at 20 weeks. Download Figure 3-1, TIF file.

10.1523/ENEURO.0453-25.2026.f3-2 Figure 3-2 ***Ogt*^N648Y/y^*Oga*^D285A/+^** **mice** **behaviour tests** Data for behaviour tests performed in *Ogt*^+/y^*Oga*^+/+^, *Ogt*^+/y^*Oga*^D285A/+^, *Ogt*^N648Y/y^*Oga*^+/+^ and *Ogt*^N648Y/y^*Oga*^D285A/+^ mice. Data are represented as mean ± SD, n = 12 for all genotypes. One-way ANOVA (alpha, 0.05) is used for statistics. Significance shown as: *p < 0.05, **p < 0.01 and ***p < 0.001. **(a)** Time to perform the T-turn of *Ogt*^+/y^*Oga*^+/+^ (black), *Ogt*^+/y^*Oga*^D285A/+^(pink), *Ogt*^N648Y/y^*Oga*^+/+^ (green) and *Ogt*^N648Y/y^*Oga*^D285A/+^ (purple) mice during the static rods test. **(b)** Delta time (total time – time to perform the T-turn) of *Ogt*^+/y^*Oga*^+/+^ (black), *Ogt*^+/y^*Oga*^D285A/+^(pink), *Ogt*^N648Y/y^*Oga*^+/+^ (green) and *Ogt*^N648Y/y^*Oga*^D285A/+^ (purple) mice during the static rods test. **(c)** Number of digging events during 3 min observation. **(d)** Time spent digging during 3 min observation. **(e)** Number of self-grooming events during 3 min observation. **(f)** Time spent self-grooming during 3 min observation. **(g)** Representative footprints of *Ogt*^+/y^*Oga*^+/+^, *Ogt*^+/y^*Oga*^D285A/+^, *Ogt*^N648Y/y^*Oga*^+/+^ and *Ogt*^N648Y/y^*Oga*^D285A/+^ mice. Red footprints correspond to the forepaws; blue footprints correspond to the hind paws. Lines represent measured distances. **(h)** Measurement of stride length as the distance between footprints of the same hind paw. **(i)** Measurement of step width as the contralateral distance between hind and front paws. **(j)** Measurement of rear paws base of support (RBOS) as the contralateral distance between hind paws. **(k)** Measurement of front paws base of support (FBOS) as the contralateral distance between forepaws. **(l)** Measurement of step alternation as the ipsilateral distance between hind and front paws. **(m)** Stride length normalised to body length. **(n)** Step width normalised to body length. **(o)** RBOS normalised to body length. **(p)** FBOS normalised to body length. **(q)** Step alternation normalised to body length. Download Figure 3-2, TIF file.

10.1523/ENEURO.0453-25.2026.f3-3 Figure 3-3 **O*g*tN648Y^/y^*Oga*^D285A/+^ blood biochemistry** Data for 20-week-old *Ogt*^+/y^*Oga*^+/+^ (n = 11), *Ogt*^+/y^*Oga*^D285A/+^ (n = 11), *Ogt*^N648Y/y^*Oga*^+/+^ (n = 12), and *Ogt*^N648Y/y^*Oga*^D285A/+^ (n = 11) mice. Data are represented as mean ± SD. One-way ANOVA (alpha, 0.05) is used for statistics. Significance shown as: *p < 0.05, **p < 0.01 and ***p < 0.001 **(a)** Quantification of *Ogt* mRNA levels in blood by RT-PCR. **(b)** Quantification of *Oga* mRNA levels in blood by RT-PCR. One *Ogt*^+/y^*Oga*^+/+^ outlier was excluded from the analysis. Download Figure 3-3, TIF file.

We then evaluated the morphological parameters of the *Ogt*^N648Y/y^*Oga*^D285A/+^ mice. Both *Ogt*^N648Y/y^ and *Ogt*^N648Y/y^*Oga*^D285A/+^ animals exhibited reduced body weight, body length, skull size, and brain weight compared with their WT and *Oga*^D285A/+^ littermates (Extended Data Fig. 3-1). No differences between *Ogt*^N648Y/y^ and *Ogt*^N648Y/y^*Oga*^D285A/+^ genotypes were observed for all measured parameters, suggesting no restoration of body morphometrics in the *Ogt*^N648Y/y^*Oga*^D285A/+^ animals. To investigate whether *Ogt*^N648Y/y^*Oga*^D285A/+^ mice will show improved behavior parameters, we performed static rods and digging tests, for which pronounced phenotypes were observed in *Ogt*^N648Y/y^ mice **(**Fig. 2*I*–*O*). We also included gait analysis to detect ataxia and gait abnormalities that have been reported in patients carrying both *Ogt*^N648Y/y^ and *Ogt*^C921Y/y^ variants (Pravata et al., 2019a; Omelková et al., 2023). Similar T-turn times were observed between both *Ogt*^N648Y/y^ and *Ogt*^N648Y/y^*Oga*^D285A/+^ genotypes during the static rods test (Extended Data Fig. 3-2*A,B*). Similarly, no differences in digging or self-grooming time and event activities were observed between *Ogt*^N648Y/y^ and *Ogt*^N648Y/y^*Oga*^D285A/+^ mice (Extended Data Fig. 3-2*C–F*). Of note, both digging and self-grooming behaviors were measured in a single 3 min period with mice placed in a cage with 5 cm bedding as opposed to panels Figure 2*N*,*O* where two separate periods and bedding heights were used. Although both *Ogt*^N648Y/y^ and *Ogt*^N648Y/y^*Oga*^D285A/+^ mice exhibited shorter stride length, narrower step width, and impaired step alternation (Extended Data Fig. 3-2*G–L*) compared with WT littermates in gait analysis, no differences were observed after normalization with body length between the four genotypes for all parameters (Extended Data Fig. 3-2*M–Q*), suggesting unaltered gait in both *Ogt*^N648Y/y^ and *Ogt*^N648Y/y^*Oga*^D285A/+^ mice.

We next examined the effect of genetic loss of OGA activity on O-GlcNAc homeostasis in the *Ogt*^N648Y/y^*Oga*^D285A/+^ mice. We assessed levels of OGT, OGA, and O-GlcNAc by Western blotting (Fig. 3*B*). OGT protein levels in the double mutant remained mostly unchanged compared with *Ogt*^N648Y/y^ mice; however, the amount of OGA protein increased (Fig. 3*C*,*D*). OGT/OGA protein ratio in *Ogt*^N648Y/y^*Oga*^D285A/+^ mice was lower compared with *Ogt*^N648Y/y^ mice (Fig. 3*E*), suggesting a partial rescue of imbalance of the O-GlcNAc cycling enzymes, although this did not affect overall O-GlcNAcylation (Fig. 3*F*). To further explore this, we measured *Ogt* and *Oga* mRNA expression in whole brains from *Ogt*^N648Y/y^*Oga*^D285A/+^ mice and their littermates, showing that *Ogt* mRNA expression is unchanged in *Ogt*^N648Y/y^*Oga*^D285A/+^ mice compared with *Ogt*^N648Y/y^, while *Oga* mRNA expression appears to be slightly increased (Fig. 3*G*,*H*). When combining both measurements, we found that the *Ogt/Oga* ratio in the brain was significantly lower in *Ogt*^N648Y/y^*Oga*^D285A/+^ mice compared with *Ogt*^N648Y/y^ littermates (Fig. 3*I*), similar to the observations at the protein level. We also analyzed *Ogt* and *Oga* mRNA expression in the blood of the same mice as a proxy of brain O-GlcNAc dyshomeostasis. Indeed, the *Ogt/Oga* ratio was also lower in blood of *Ogt*^N648Y/y^*Oga*^D285A/+^ mice compared with *Ogt*^N648Y/y^ littermates (Fig. 3*J*, Extended Data Fig. 3-3). Overall, these findings suggest that although we did not observe rescue of morphological and behavioral deficits in the *Ogt*^N648Y/y^*Oga*^D285A/+^ mice, genetic inhibition of OGA did achieve partial rescue of brain O-GlcNAc dyshomeostasis that can be indirectly measured as a biomarker in peripheral blood.

## Discussion

Pathogenic variants in OGT have been linked to ID, resulting in the recently described OGT-CDG syndrome (Pravata et al., 2020; Omelková et al., 2023; Authier et al., 2024; Mayfield et al., 2024). Affected individuals exhibit a wide range of physical, neurological, and behavioral symptoms, with varying degrees of prevalence and severity. OGT-CDG variants are mostly de novo mutations found in families worldwide, contributing to the heterogeneity of the disorder. Animal models are valuable for studying disease etiology, uncovering underlying mechanisms, and developing treatments. Therefore, to dissect the molecular basis of OGT-CDG pathology and establish a platform for evaluating possible treatments of the disease, vertebrate OGT-CDG models are required to establish patient-linked, reliably quantifiable phenotypes. Due to the heterogeneity in symptoms and the number of reported variants, multiple models are necessary to fully recapitulate the disease and expand the identification of phenotypes for targeted therapeutic interventions. Previously, we successfully generated and described a mouse carrying an OGT-CDG variant in the catalytic core, *Ogt*^C921Y/y^ (Authier et al., 2024, 2025), which shows altered O-GlcNAc homeostasis, reduced body and brain size, and behavioral abnormalities, including hyperactivity and impulsivity. In the present study, we provided a biochemical, morphological, and behavioral characterization of a newly generated mouse line with the N648Y variant in the catalytic core of OGT and used a genetic approach to correct O-GlcNAc dyshomeostasis.

The OGT^N648Y^ variant is a de novo variant in *OGT* found in the second child of an Estonian nonconsanguineous couple (Pravata et al., 2019a). The patient exhibited a range of symptoms similar to those found in other OGT-CDG patients, such as intellectual disability, dysmorphic features, and developmental delay (Pravata et al., 2020). *Ogt*^N648Y/y^ mice exhibit characteristic morphological features such as lower body weight and size, also often observed in other mouse models of X-linked IDs, where the mice show decreased body weight but remain healthy and fertile (Iwase et al., 2016). *Ogt*^N648Y/y^ mouse skulls are smaller in size and differ in shape compared with WT. A number of OGT-CDG patients show skull asymmetry and microcephaly (Pravata et al., 2019a, 2020), clinically described as a reduction in the head circumference, which suggests a decrease in brain volume (Woods, 2004). *Ogt*^N648Y/y^ mice display a microcephaly phenotype that is stronger than that observed in the other OGT-CDG line currently available (Authier et al., 2024, 2025), and the brains of *Ogt*^N648Y/y^ mice are smaller than those of WT mice. However, as opposed to *Ogt*^C921Y/y^ mice, most of the regions in the *Ogt*^N648Y/y^ adult mice are globally reduced and scaled with the total brain volume, with no regions differentially smaller after multiple-comparisons correction. *Ogt*^N648Y/y^ mice behave differently from WT mice in the static rods test, particularly regarding the performance of the T-turn, which suggests defects in motor coordination and balance. Furthermore, they show reduced digging activity, while other tests assessing compulsive behavior are not affected. The previously described *Ogt*^C921Y/y^ mice exhibited similar impairments in the static rod tests and digging activity (Authier et al., 2025). In other mouse models of IDs and autism spectrum disorders, a decrease in the digging activities was also observed, together with an increase in self-grooming (Wang et al., 2011; Won et al., 2012; M. Yang et al., 2012; Oaks et al., 2017). The decrease in free digging indicates that OGT-CDG variants affect natural mouse behavior (de Brouwer et al., 2019) and are not likely to be due to changes in anxiety levels as time spent in open arms and light compartment were comparable between WT and OGT-CDG mice in EPM and dark/light tests, respectively. Therefore, *Ogt*^N648Y/y^ mice exhibit motor defects and autistic spectrum disorder-related behaviors, features that recapitulate patients' characteristics (Mayfield et al., 2024). Notably, during OF, EPM, and dark/light tests, the *Ogt*^N648Y/y^ mice show a tendency to increase the distance traveled, which may indicate mild hyperactivity, a characteristic also reported for the patient carrying the OGT^N648Y^ variant and more evident in the previously described *Ogt*^C921Y/y^ mice (Authier et al., 2025). Regarding cognitive tasks, the *Ogt*^N648Y/y^ mice showed comparable abilities to discriminate a novel from a familiar object in the NOR task. This was also observed in the *Ogt*^C921Y/y^ mice together with similar spontaneous alternation performance in the T maze (Authier et al., 2025), suggesting intact memory abilities in OGT-CDG mice. In addition, while similar freezing behavior was observed 24 h post aversive conditioning in *Ogt*^C921Y/y^ mice compared with WT control, the *Ogt*^C921Y/y^ mice showed significant delay in freezing during the acquisition phase (Authier et al., 2025), indicative of a learning deficit in these mice. Further investigations using more challenging learning paradigms will be necessary to expand the characterization of OGT-CDG mouse models and investigate how different variants in the OGT gene causing ID impact learning processes. Taken together, this work establishes quantifiable morphometric and behavioral phenotypes in an OGT-CDG mouse model that can be used to dissect mechanisms and evaluate future treatments.

To date, several mechanisms have been proposed to explain the disease, including disruptions in O-GlcNAc homeostasis, HCF1 processing, or misfolding of the OGT protein (Pravata et al., 2020). *Ogt*^N648Y/y^ mice exhibit reduced global O-GlcNAc levels in the brain, in agreement with what was observed in mouse embryonic stem cells carrying the mutation (Pravata et al., 2019a) and in other models carrying the OGT^C921Y^ variant (Omelková et al., 2023; Authier et al., 2024; Czajewski et al., 2024). Both OGT and OGA protein levels appear to be lower in the brains of the *Ogt*^N648Y/y^ mouse, but the reduction in OGT protein levels is not explained by reduced transcription, as *Ogt* mRNA levels are, in fact, increased, presumably as a compensatory mechanism. X-ray crystallography performed with the OGT^N648Y^ variant indicates structural rearrangements in the catalytic domain, suggesting that OGT protein folding or stability may be affected (Pravata et al., 2019a). These observations, along with the reduction in *Oga* mRNA levels, were likewise observed in other OGT-CDG mice (Authier et al., 2025) and suggest a compensatory mechanism to counteract the pathogenic hypoglycosylation. Taken together, these findings suggest O-GlcNAc dyshomeostasis as a common hallmark in OGT-CDG mice.

Several neurological diseases are linked to disrupted O-GlcNAc balance, and there are multiple cases where OGA inhibition has been suggested as a possible treatment approach (Yuzwa et al., 2014; Hastings et al., 2017; Hsieh et al., 2019; Sanchez et al., 2019; Tavassoly et al., 2021; Lanzillotta et al., 2025). Here, we use a genetic approach to restore O-GlcNAc levels in *Ogt*^N648Y/y^ mice using *Oga*^D285A/+^ mice, aiming to mimic chronic OGA inhibition from embryogenesis to adulthood in the context of OGT-CDG. *Oga*^D285A/+^ mice exhibit increased OGA levels and reduced OGT protein expression, likely as a response to the deficiency in OGA catalytic activity (Muha et al., 2021). Meanwhile, total O-GlcNAc levels remain elevated due to the reduction of OGA activity. As homozygous *Oga*^D285A/D285A^ mice do not survive after birth, we used heterozygous *Oga*^D285A/+^ mice for modulating O-GlcNAc homeostasis in *Ogt*^N648Y/y^ mice. Heterozygous *Oga*^D285A/+^ mice develop normally (Muha et al., 2021), and in agreement with this, we show here that heterozygous *Oga*^D285A/+^ mice display similar morphometric parameters and behavior to wild-type mice in all tests conducted, suggesting that moderate, chronic, and longitudinal elevation of O-GlcNAcylation levels is in principle a safe approach for OGT-CDG phenotypic rescue. While in the absence of a specific stressor or pathology, elevated O-GlcNAc may interfere with normal neuronal function (Parween et al., 2017; Yang et al., 2017), an increase in O-GlcNAc levels has been shown to provide a neuroprotective effect against diseased or aging brains (Yuzwa et al., 2012; Wheatley et al., 2019; Lee et al., 2020). In *Ogt*^N648Y/y^*Oga*^D285A/+^ mouse brains, we achieved a partial rescue of OGT/OGA homeostasis, driven by an increase in OGA protein levels and mRNA expression in response to heterozygotic loss of OGA activity. However, the partial correction of the OGT/OGA ratio was not sufficient to restore O-GlcNAc levels, behavioral deficits, and morphometrics in these mice. The use of heterozygous mice due to homozygotic lethality constitutes one limitation of our approach, as heterozygous mice still produce a functional copy of OGA, which may compensate for the heterozygotic loss of OGA function. Previous studies indicated that it is necessary to inhibit OGA by >80% to observe neuroprotective hyper-O-GlcNAcylation in the brains of mouse models of neurodegenerative diseases (Yuzwa et al., 2014; Hastings et al., 2017; Kielbasa et al., 2024). Although our genetic approach did not rescue OGT-CDG phenotypes, it demonstrates the validity of OGA targeting in modulating OGT/OGA homeostasis in OGT-CDG from conception without leading to deleterious effects. Future avenues for modulating O-GlcNAc homeostasis in OGT-CDG mice may include the use of small molecules to achieve greater OGA inhibition and metabolic approaches. Recently, nutrient supplementation in different CDG models has been studied as a treatment option (Witters et al., 2021; Boyer et al., 2022; den Hollander et al., 2023; Jáñez Pedrayes et al., 2025), suggesting that using sugars that increase protein O-GlcNAcylation may be beneficial in the context of OGT-CDG. Similarly, supplementation with GlcNAc itself has recently been shown to have beneficial effects in multiple sclerosis patients in a small clinical trial (Sy et al., 2023). Another metabolite of interest is glucosamine, which can enter the hexosamine biosynthetic pathway, leading to increased UDP-GlcNAc levels and, consequently, directly boost protein O-GlcNAc levels (Swamy et al., 2016; Hu et al., 2017). Glucosamine is a widely used supplement for joint health (Black et al., 2009; Ogata et al., 2018), and it has also been suggested to be beneficial in several metabolic diseases (Li et al., 2023; Ryu et al., 2025). Supplementation with glucosamine has already been explored in the patient carrying the OGT^N648Y^ variant and may have a positive impact without any adverse effects (Pravata et al., 2019a). OGT-CDG mice may serve as a crucial model to better understand the impact of metabolite supplementation in the context of this disease.

Biomarkers serve as measurable and accessible indicators of both disease traits and the impact of a treatment or intervention. In OGT-CDG, such biomarkers are urgently needed to evaluate the effect of genetic or pharmacological rescue strategies. Recently, O-GlcNAc dyshomeostasis has been proposed as a biomarker for identifying new pathogenic OGT-CDG variants using a stem cell reporter line (Yuan, Ferenbach, et al., 2025; Yuan, Mitchell, et al., 2025). Importantly, peripheral blood has been used to identify O-GlcNAc-related changes in both disease and treatment contexts. In a study involving an OGA inhibitor as treatment for proteinopathies, changes in protein O-GlcNAcylation were detected in both brain and blood (Permanne et al., 2022). Similarly, in patients with diabetes mellitus, OGA expression and activity in leukocytes correlated with systemic inflammation (Pagesy et al., 2019). Here, we observed a partial rescue of *Ogt/Oga* mRNA ratio in the brain that was also detectable in leukocytes extracted from peripheral blood. These findings highlight the potential of using blood *Ogt*/*Oga* mRNA ratio not only for OGT-CDG pathogenicity detection but also as a biomarker to evaluate treatment-induced modulation of O-GlcNAc dyshomeostasis in OGT-CDG disorder.

In conclusion, the *Ogt*^N648Y/y^ mouse line recapitulates key features of OGT-CDG, including disrupted O-GlcNAc homeostasis, growth deficits, and behavioral changes, making it a valuable model for dissecting disease mechanisms. Although the genetic rescue approach used here only partially restored O-GlcNAc balance in the brain without improving behavior or morphology, the molecular changes were also observed in peripheral blood, supporting its use as an accessible biomarker for the disease and treatment response. Future research will focus on exploiting these OGT-CDG mouse models and biomarkers to evaluate therapeutic strategies for this patient population with unmet medical need.

## Synthesis

Reviewing Editor: Fabienne Poulain, University of South Carolina Decisions are customarily a result of the Reviewing Editor and the peer reviewers coming together and discussing their recommendations until a consensus is reached. When revisions are invited, a fact-based synthesis statement explaining their decision and outlining what is needed to prepare a revision will be listed below. The following reviewer(s) agreed to reveal their identity: Chad Slawson. Note: If this manuscript was transferred from JNeurosci and a decision was made to accept the manuscript without peer review, a synthesis may not be available.

This study presents a novel mouse model of OGT-CDG (OgtN648Y/y), a human congenital disorder of glycosylation due to mutations in the OGT enzyme that adds O-GlcNAc to proteins. The authors demonstrate that the OgtN648Y/y mouse model recapitulates some of the symptoms found in OGT-CDG patients including microcephaly, sensory-motor deficits, and brain O-GlcNAc dyshomeostasis. Crossing the OgtN648Y/y mouse model with the OgaD285A mouse that expresses an inactive form of O-GlcNAcase (OGA), the enzyme responsible for removing protein O-GlcNAcylation, leads to a partial rescue of brain O-GlcNAc dyshomeostasis homeostasis but does not rescue the PGT-CDG morphological and behavioral deficits. Overall, the manuscript is well written and presents an interesting novel mouse model of OGT-CDG that can be exploited in the future for identifying biomarkers of OGT-CDG and testing therapeutic approaches. The authors need to address the following points before this manuscript can be considered for publication in eNeuro:

1) A general concern relates to the observation that the defects present in the mouse differ from the human clinical phenotype. The authors identified motor impairments in the OGT-CDG mice but only very mild, if any, effects on anxiety, and no clear effects on cognitive behaviors. In contrast, patient symptoms are quite severe. Are there changes in anxiety levels at all? The results in Fig 2K-O may indicate elevated levels of anxiety but may also be due to motor skill problems shown in other figures. Similarly, Fig S3J-M may indicate increased anxiety but as the time spent in the light area is not changed, observing more light area entries may be a factor of the increased motor behavior indicated in Fig S3A-C. In addition, the putative anxiety phenotype seems quite unstable as the change in self-grooming time seen in Fig 2N is not seen in Fig S5C between the OGT mutant and wildtypes. More problematic, other anxiety tests do not indicate clearly that there are changes in anxiety levels. Would it be possible to do more anxiety tests that do not rely on motor function to further clarify the phenotype? Similarly, more tests on cognitive function should be completed to better judge what the cognitive effects are of this mutation that is supposed to affect cognitive function.

2) It is surprising (and worth reporting) that the mouse lines can show such weak phenotypes when the changes in OGT, OGA and O-GlcNAc levels are (at least for the OGT-CDG mutant) dramatically changed. Is there another mechanism than altered O-GlcNAcylation that drives the phenotype?

Minor points:

1) Fig S1 and Fig S2 would be easier to read if they were presented in a table format.

2) It's unclear what comparisons are significant in Fig S3C. Is the maximum speed in Day 3 higher in mutants than in wildtypes?

3) Fig S3D: the result for the mutants with 10 mm bar seem to be driven by two extreme outliers. Is it appropriate to include these in the analyses? Do the mutants take significantly longer time on the 10 mm bar?

4) The following statement should be revised: "Moreover, an increase in O-GlcNAc levels has been shown not to affect the structure and function of neurons and to provide a neuroprotective effect against diseased or aging brains (33,37,69)". Increased O-GlcNAc has been shown to negatively affect neuron function (for example, see Yang et al. in Scientific reports, 2017).

5) It says that none of the regional differences in brain area volumes are significant after multiple comparisons corrections. However, figures S1 and S2 present un-corrected and significant statistics, which is misleading.

## Author Response

Synthesis of Reviews:

Computational Neuroscience Model Code Accessibility Comments for Author (Required):N/A Synthesis Statement for Author (Required):

This study presents a novel mouse model of OGT-CDG (OgtN648Y/y), a human congenital disorder of glycosylation due to mutations in the OGT enzyme that adds O-GlcNAc to proteins. The authors demonstrate that the OgtN648Y/y mouse model recapitulates some of the symptoms found in OGT-CDG patients including microcephaly, sensory-motor deficits, and brain O-GlcNAc dyshomeostasis. Crossing the OgtN648Y/y mouse model with the OgaD285A mouse that expresses an inactive form of O-GlcNAcase (OGA), the enzyme responsible for removing protein O-GlcNAcylation, leads to a partial rescue of brain O-GlcNAc dyshomeostasis homeostasis but does not rescue the PGT-CDG morphological and behavioral deficits. Overall, the manuscript is well written and presents an interesting novel mouse model of OGT-CDG that can be exploited in the future for identifying biomarkers of OGT-CDG and testing therapeutic approaches. The authors need to address the following points before this manuscript can be considered for publication in eNeuro:

1) A general concern relates to the observation that the defects present in the mouse differ from the human clinical phenotype. The authors identified motor impairments in the OGT-CDG mice but only very mild, if any, effects on anxiety, and no clear effects on cognitive behaviors. In contrast, patient symptoms are quite severe. Are there changes in anxiety levels at all? The results in Fig 2K-O may indicate elevated levels of anxiety but may also be due to motor skill problems shown in other figures. Similarly, Fig S3J-M may indicate increased anxiety but as the time spent in the light area is not changed, observing more light area entries may be a factor of the increased motor behavior indicated in Fig S3A-C. In addition, the putative anxiety phenotype seems quite unstable as the change in self-grooming time seen in Fig 2N is not seen in Fig S5C between the OGT mutant and wildtypes. More problematic, other anxiety tests do not indicate clearly that there are changes in anxiety levels. Would it be possible to do more anxiety tests that do not rely on motor function to further clarify the phenotype? Similarly, more tests on cognitive function should be completed to better judge what the cognitive effects are of this mutation that is supposed to affect cognitive function.

We thank the reviewers for their time and effort in improving the quality of our manuscript. OGT-CDG is a newly described syndrome with only a few cases reported, for which the phenotype-genotype correlation has not been fully assessed. From the published pathogenic OGT variants collection (currently at 13), it is becoming apparent that OGT-CDG represents an heterogenous syndrome varying from mild to severe ID, developmental delay and peripheral tissues comorbidities. The level of heterogeneity can have several origins, including genetic background of the patients and/or differential pathogenic severity of each individual OGT-CDG variant. It is therefore unlikely that a single animal model will ever fully recapitulate all of the clinical symptoms, even without considering that a mouse is not a human! Nevertheless, the OgtN648Y/y mice (constituting the second ever mouse model generated to carry OGT-CDG pathogenic variants), showed morphological and behaviour deficits including hyperactivity and motor impairments remiscent of some of the OGT-CDG patients symptoms, making it a valuable tool to better understand the disease and underlined mechanisms.

Regarding a putative anxiety behaviour, OgtN648Y/y mice show similar time spent in open/closed arms during Elevated Plus Maze (EPM) (Fig. S3F,I, now renamed as Extended Data Fig. 2_5F,I to match the journal's specifications) and similar time spent in the light compartment during the dark/light test (Extended Data Fig. 2_5M) compared to WT animals. These two key parameters suggest similar anxiety levels between WT and OgtN648Y/y mice. Therefore we agree with the reviewer that the increase in center entries in EPM (Extended Data Fig. 2_5H), light entries in dark/light (Extended Data Fig. 2_5L) and distance in both EPM (Extended Data Fig. 2_5G) and dark/light (Extended Data Fig. 2_5K) observed are suggestive of a hyperlocomotion phenotype in the OgtN648Y/y mice compared to WT rather than anxiety behaviour. We would also like to point out that the digging and self-grooming tests presented in Fig. 2K-O were used to assess compulsive and repetitive behaviours, features that can be observed in some of the patients with OGT-CDG variants rather than anxiety levels for which EPM and dark-light paradigms constitute gold standard approaches. The discussion has been adjusted to clarify these points.

We acknowledge that it was not indicated in original manuscript that self-grooming assessment in Fig 2N and O and Fig. S5E and F (now Extended Data Fig. 3_2E and F to match journal's specifications) were not performed in the same settings. For the characterisation of the OgtN648Y/y line alone, digging and self-grooming behaviours were measured in two separate 3 min observation periods with mice placed in a cage with 5 cm bedding (to promote natural digging behaviour) for digging assessment and 1 cm bedding for self-groowing evaluation. As opposed to panel Extended Data Fig. 3_2C-F where a single 3 min period observation was used with mice placed in a cage with 5 cm bedding to both assess digging and self grooming behaviours in the crossing of OgaD285N/+ with OgtN648Y/y mice and their controls. Thus a direct comparison of the results presented in Fig 2N and O and Extended Data Fig. 3_2E and F is not possible. The result section has been adjusted to clarify these points.

We agree with the reviewers that our already very large battery of behavioral tests does not fully cover possible cognitive tests. The main aim of the manuscript was to describe a new OGT-CDG mouse model with patient-relevant phenotypes and a first attempt at rescuing these. Given that the approach of crossing OgaD285A/+ with OgtN648Y/y mice does not lead to behaviour deficit rescue, the addition of other tests will fall beyond the scope of this manuscript. The discussion has been adjusted to highlight the limitation of our behavioural screens in regards to cognition assessment in the OgtN648Y/y mouse line. Nevertheless, we appreciate the reviewers' comment and will continue to work along these lines, and report in a follow up manuscript in the future.

2) It is surprising (and worth reporting) that the mouse lines can show such weak phenotypes when the changes in OGT, OGA and O-GlcNAc levels are (at least for the OGT-CDG mutant) dramatically changed. Is there another mechanism than altered O-GlcNAcylation that drives the phenotype? It is not known yet whether OGT-CDG is caused by loss of O-GlcNAc modification on specific proteins or due to other possible mechanisms (e.g. OGT interactome, HCF1 processing, feedback on OGA levels) which did not intend to investigate in the current manuscript, which is focused on identification of phenotypes and a genetic attempt at rescue thereof. The compensatory reduction in OGA levels to maintain minimal O-GlcNAc levels possibly account for milder phenotypes compared to OGT/OGA knock out models where total protein loss and associated severe O-GlcNAc dyshomeostais lead to lethality. Instead, our OgtN648Y/y model shows quantifiable morphological and behaviour deficits associated with O-GlcNAc dyshomeostasis and these mice can now serve as a tool to investigate underlying mechanisms that drive the observed phenotypes. We appreciate the reviewers' comment and will continue to work along these lines, and report in a follow up manuscript in the future.

Minor points:

1) Fig S1 and Fig S2 would be easier to read if they were presented in a table format.

We agree with the reviewers that Supplementary Figures S1 and S2 attempted to convey too much information and were therefore difficult to read. Following the reviewer's suggestion, we have summarized the key information from Figures S1 and S2 (now Extended Data 2_1 and 2_3) in two tables (Extended Data 2_2 and 2_4), which now report only whether p-values corrected for multiple comparisons were below or above an alpha of 0.05. This improves clarity and aligns the supplementary material more closely with the main conclusions of the manuscript.

2) It's unclear what comparisons are significant in Fig S3C. Is the maximum speed in Day 3 higher in mutants than in wildtypes? We agree that, as presented, it is unclear which significant comparisons are indicated in figure S3C (now Extended Data Fig. 2_5C to match journal specifications). In the open field test, there is no difference in maximum speed or total distance traveled between the two genotypes. The significant differences shown in the figure reflect comparisons within the same genotype across days, which may not have been immediately apparent from the figure presentation. Therefore, the stated differences are now further detailed as follows: significant differences in Ogt+/y mice between days are indicated by a section sign ({section sign}), while differences among the OgtN648Y/y mice between days are indicated by a pound symbol (#). The figure legend was also modified to clarify these comparisons.

3) Fig S3D: the result for the mutants with 10 mm bar seem to be driven by two extreme outliers. Is it appropriate to include these in the analyses? Do the mutants take significantly longer time on the 10 mm bar? For the static rod test, a cut-off of 120 seconds was applied to a mouse that falls or does an upside-down maneuver on the bar. In Fig. S3D (now Extended Data Fig. 2_5D to match journal specifications), the two 120-sec data points with the 10 mm bar represent one mouse falling from the bar while performing the t turn, and another mouse performing an upside-down maneuver after the t turn. The Dixon's Q test was performed for outlier identification in the static rods test for the OgtN648Y/y group according to Rorabacher (https://doi.org/10.1021/ac00002a010). The experimental Q value (Qexp) was calculated from raw data using Qexp = (x2 − x1)/(xn − x1), where x1 is the value of interest, x2, the closest value to x1 in the data set, and xn the highest value in the data set. Qexp was next compared with the Q critical value (Qcrit) of 0.426 for n = 12 for a confidence level of 95 % using the Q-table. Data that displayed a Qexp higher than the Qcrit of 0.426 can be considered as an outlier at a confidence level of 95 %. Although the two data points of 120 seconds can be considered as outliers according to the Rorabacher's method (Qexp equal to 86 therefore above 0.426), we believe that removing them from the analysis won't actually reflect the overall performance of the OgtN648Y/y mutant group during the rotarod test. When removed, the time remained higher in the OgtN648Y/y group than in the WT group, with a significance level (p = 0.0197; 2-way ANOVA followed by Tuckey correction), suggesting that even the OgtN648Y/y mice that perform below the cut off of 120 seconds do need more time to perform the test.

4) The following statement should be revised: "Moreover, an increase in O-GlcNAc levels has been shown not to affect the structure and function of neurons and to provide a neuroprotective effect against diseased or aging brains (33,37,69)". Increased O-GlcNAc has been shown to negatively affect neuron function (for example, see Yang et al. in Scientific reports, 2017).

We appreciate the reviewer's comment and the opportunity to clarify this point in the manuscript. The study that highlights the negative effects of increased O-GlcNAcylation in neurons was referenced in the introduction; however, to clarify its relevance, we have added an additional sentence in the Discussion section that explicitly highlights it and cites further evidence of a possible negative effect of high O-GlcNAc levels in the brain. Our goal was to emphasize the potential beneficial effects of increasing O-GlcNAc levels in aged or diseased brains, and the text has been revised accordingly to better reflect this.

5) It says that none of the regional differences in brain area volumes are significant after multiple comparisons corrections. However, figures S1 and S2 present un-corrected and significant statistics, which is misleading.

We thank the reviewers for pointing out the potential for confusion arising from the presentation of uncorrected significant results in Supplementary Figures S1 and S2.

The original intent of including both uncorrected and corrected p-values was to provide transparency and to illustrate expected effect sizes for future power calculations.

As we believe the figures are still valuable for illustrating this, we have revised Supplementary Figures S1 and S2 (now Extended Data 2_1 and 2_3) to present only group medians and associated statistics, with a clear distinction between uncorrected and multiple-comparison-corrected results. In addition, the primary statistical inferences are now confined to the corrected results summarized in the supplementary tables (mentioned above), ensuring consistency between the text and supplementary material.
