# Supplementary figures and images for: Genetic Rescue of Pathogenic O-GlcNAc Dyshomeostasis Associated with Microcephaly and Motor Deficits
Source: eNeuro. 2026 Jun 4;13(6):ENEURO.0453-25.2026. doi: 10.1523/ENEURO.0453-25.2026 (PMC13240978; doi:10.1523/ENEURO.0453-25.2026)

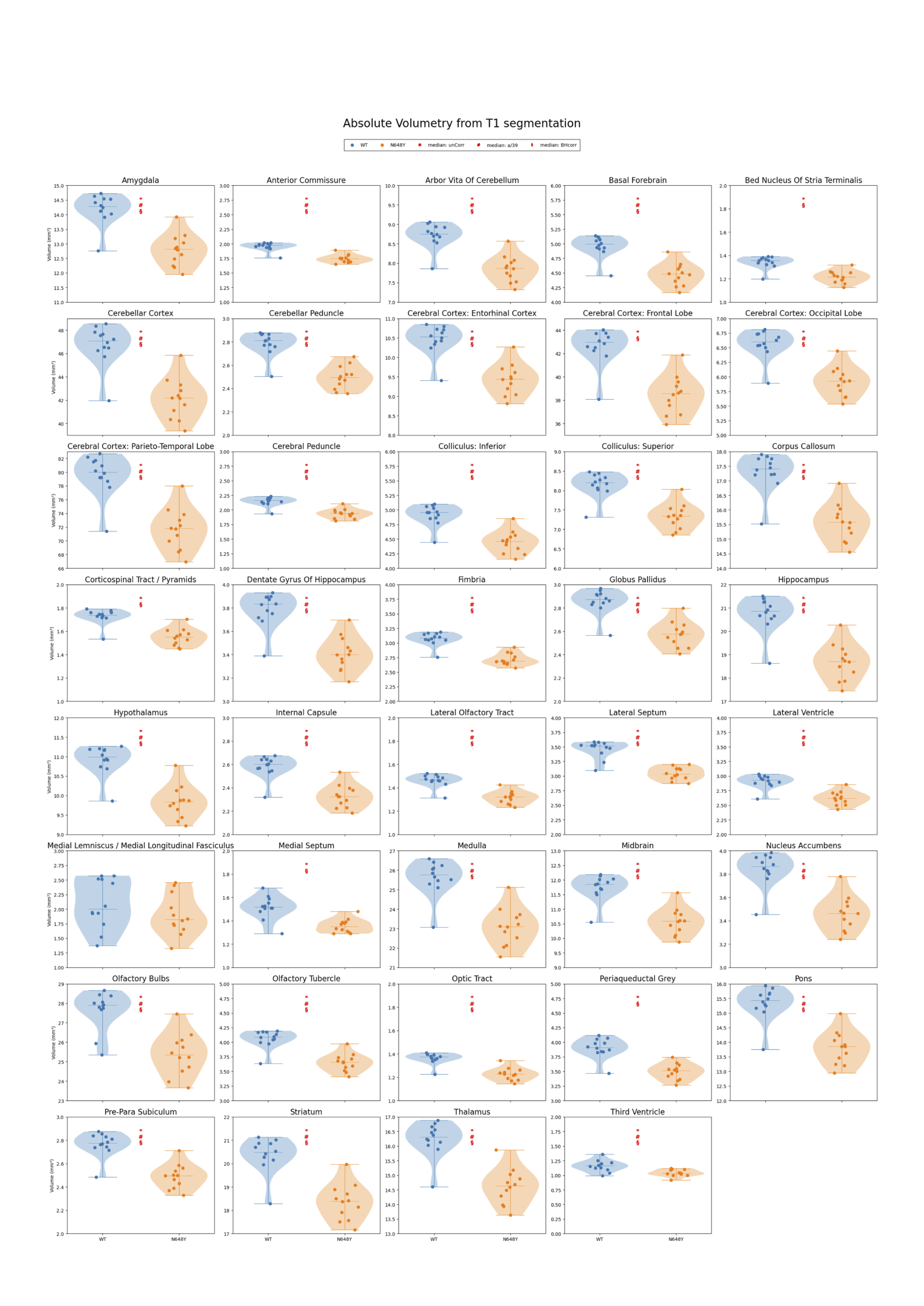

Supplement: Figure 2-1 — Absolute volumetry of 39 brain regions from T1. Each panel corresponds to a different bilaterally pooled region. Statistical significance (p < 0.05) from permutation tests (100k for each region) is indicated by asterisks (*; green for group mean, red for median) for uncorrected p-values. Correction for multiple comparisons was performed by division by the number of regions and is indicated by a pound symbol (#) or adjusted for false discovery rate (Benjamini-Hochsberg, BHcorr), indicated by a section sign (§) - if corrected, p < 0.05. Download Figure 2-1, TIF file. [file eneuro-13-ENEURO.0453-25.2026-s001.tif]

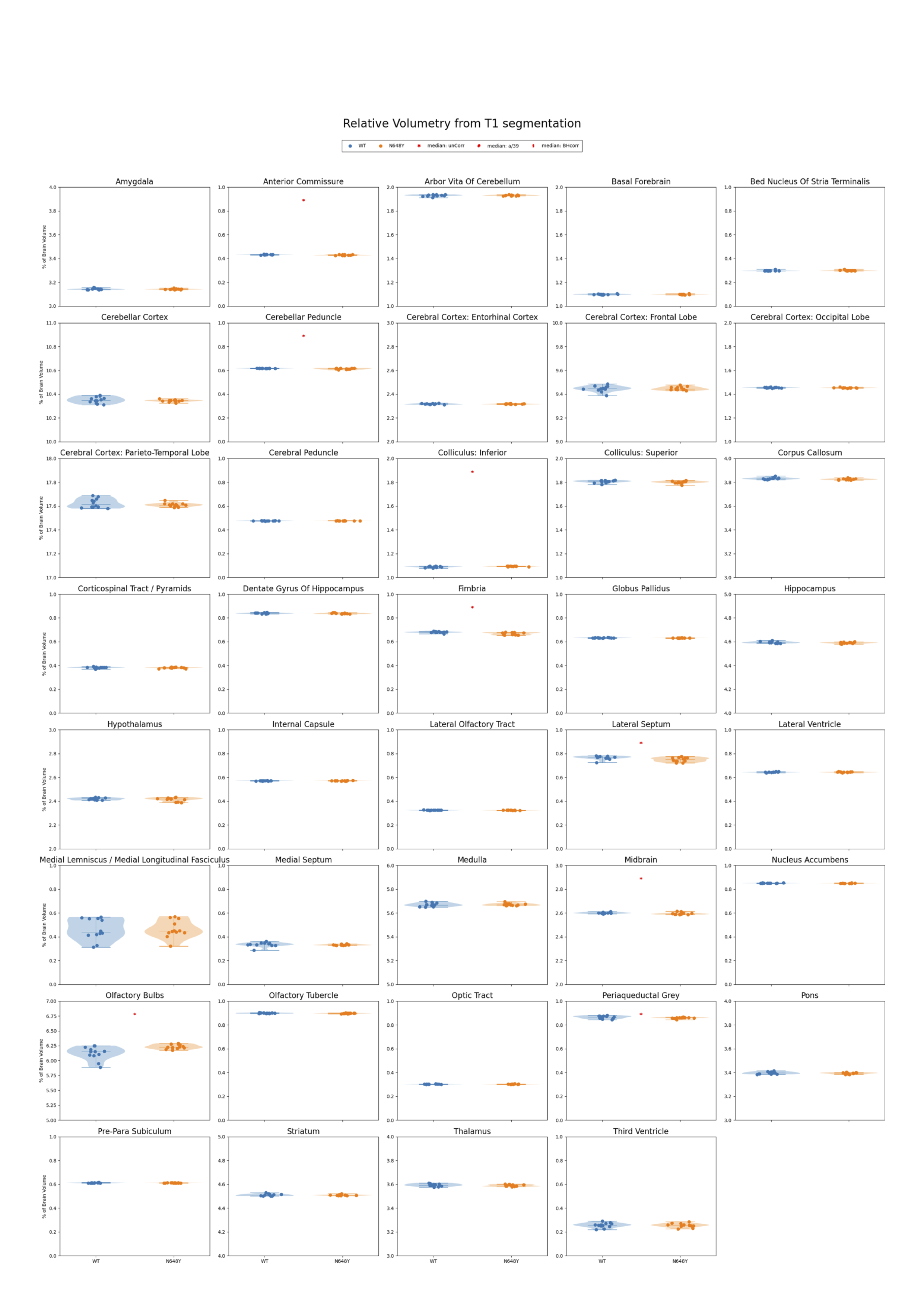

Supplement: Figure 2-3 — Relative volumetry of 39 brain regions from T1. Each panel corresponds to a different bilaterally pooled region. The regional relative volume (RRV) is normalized to the individual total brain volume. Each dot corresponds to a subject. Statistical significance (p < 0.05) from permutation tests (100k for each region) is indicated by asterisks (*; green for group mean, red for median) for uncorrected p-values. Correction for multiple comparisons was performed by division by the number of regions and is indicated by a pound symbol (#) or adjusted for false discovery rate (Benjamini-Hochsberg, BHcorr) indicated by a section sign (§) - if corrected p < 0.05. Download Figure 2-3, TIF file. [file eneuro-13-ENEURO.0453-25.2026-s003.tif]

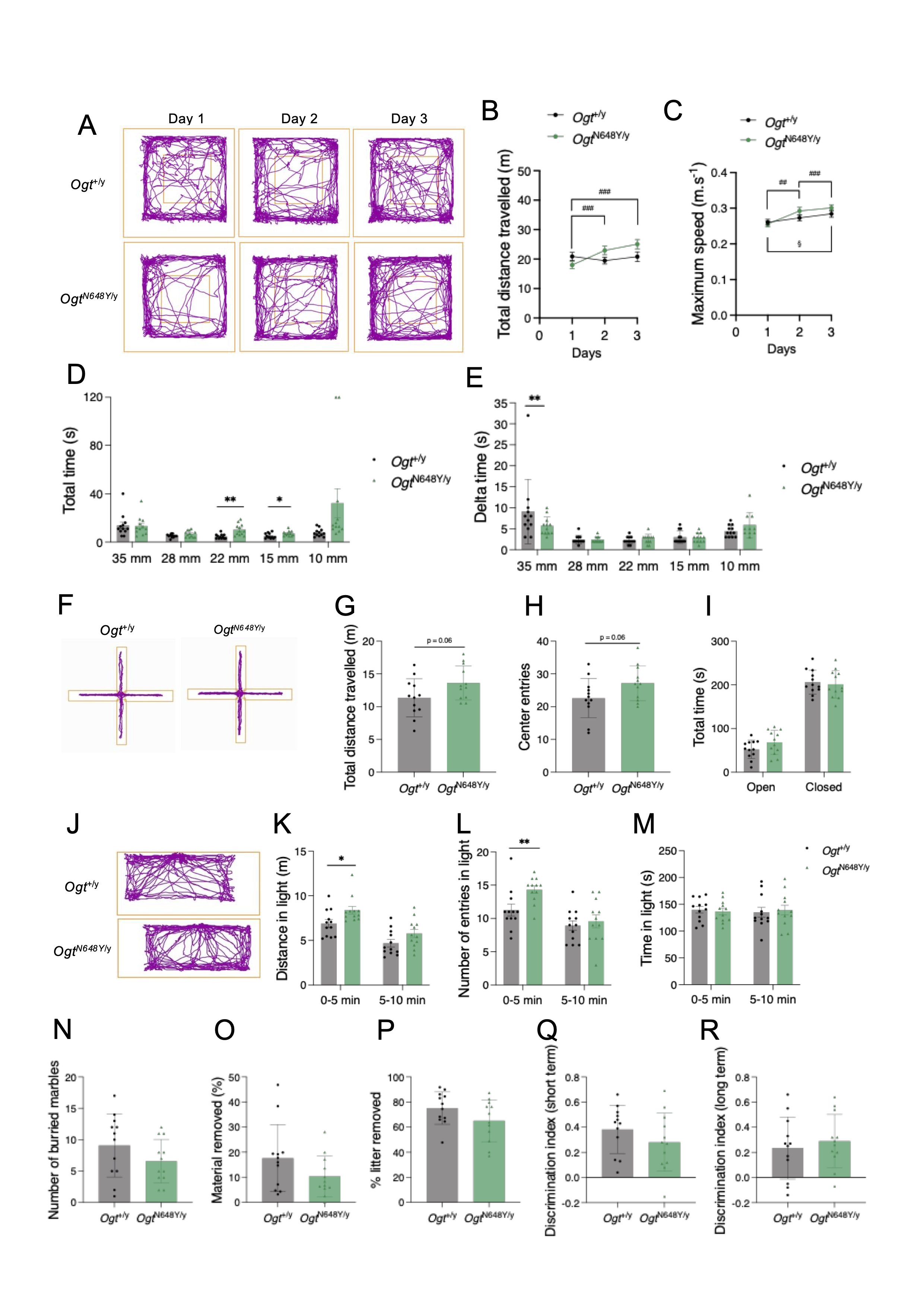

Supplement: Figure 2-5 — Behaviour of OgtN648Y/y mice. Data are represented as mean ± SD, n = 12 for all genotypes. Student’s t-test is used for statistics unless another test is indicated. Significance shown as: *p < 0.05, **p < 0.01 and ***p < 0.001 (a) Representative tracking plot of one Ogt+/y and one OgtN648Y/y mouse over three days in the open field arena (b) Total distance travelled over three consecutive days in the open field arena of Ogt+/y (black) and OgtN648Y/y (green) mice. Two-way ANOVA (alpha, 0.05) was used for statistics. Differences within Ogt+/y mice over the days are indicated by a section sign (§), while differences among the OgtN648Y/y are indicated by a pound symbol (#). (c) Maximum speed achieved over three consecutive days in the open field arena by Ogt+/y (black) and OgtN648Y/y (green) mice. Two-way ANOVA (alpha, 0.05) was used for statistics. Differences within Ogt+/y mice over the days are indicated by a section sign (§), while differences among the OgtN648Y/y are indicated by a pound symbol (#). (d) Total time to perform the static rods tests of Ogt+/y (black) and OgtN648Y/y (green) mice. (e) Delta time (total time – time to perform the T-turn) of Ogt+/y (black) and OgtN648Y/y (green) mice during the static rods test. (f) Representative tracking plot of one Ogt+/y and one OgtN648Y/y mouse during the elevated plus maze (EPM) test. (g) Total distance travelled of Ogt+/y and OgtN648Y/y mice during the EPM test. (h) Number of entries to the centre of Ogt+/y and OgtN648Y/y mice during the EPM test. (i) Total time spent in open and closed arms of Ogt+/y (black) and OgtN648Y/y (green) mice during the EPM test. (j) Representative tracking plot in the light compartment of one Ogt+/y and one OgtN648Y/y mouse during the dark/light paradigm. (k) Distance travelled in the light of Ogt+/y (black) and OgtN648Y/y (green) mice during the dark/light test. (l) Number of entries in the light of Ogt+/y (black) and OgtN648Y/y (green) mice during the dark/light paradigm. (m) Time spen [file eneuro-13-ENEURO.0453-25.2026-s005.tif]

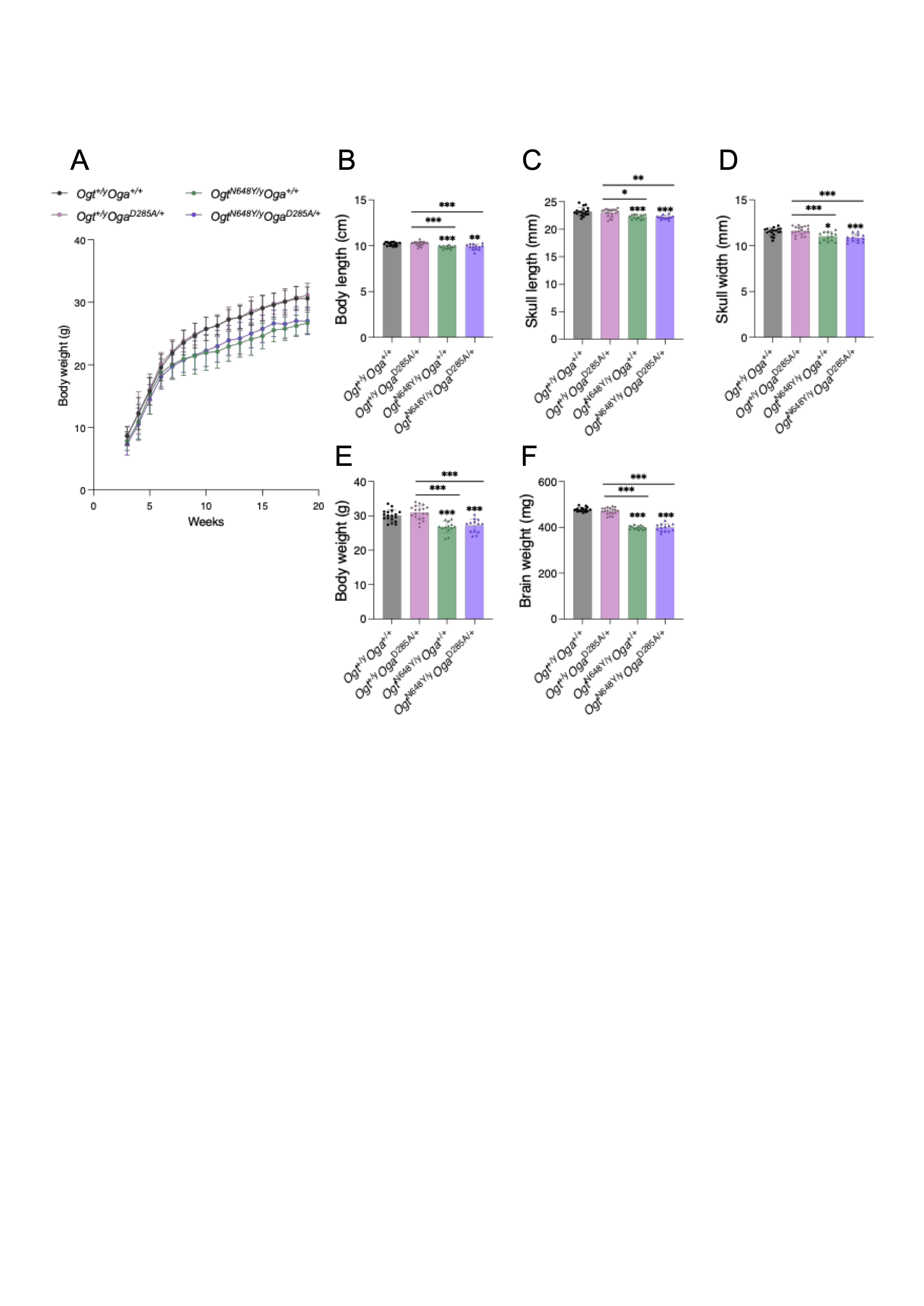

Supplement: Figure 3-1 — OgtN648Y/yOgaD285A/+ mice morphometrics Data for 20-week-old Ogt+/yOga+/+, Ogt+/yOgaD285A/+, OgtN648Y/yOga+/+ and OgtN648Y/yOgaD285A/+ mice unless otherwise stated. Data are represented as mean ± SD, n = 12 for all genotypes. One-way ANOVA (alpha, 0.05) is used for statistics unless another test is indicated. Significance shown as: *p < 0.05, **p < 0.01 and ***p < 0.001. (a) Measurement of body weight over time. Two-way ANOVA (alpha, 0.05) is used for statistics. (b) Measurement of body length at 20 weeks. (c) Measurement of skull length at 20 weeks. (d) Measurement of skull width at 20 weeks. (e) Measurement of body weight at 20 weeks (f) Measurement of brain weight at 20 weeks. Download Figure 3-1, TIF file. [file eneuro-13-ENEURO.0453-25.2026-s006.tif]

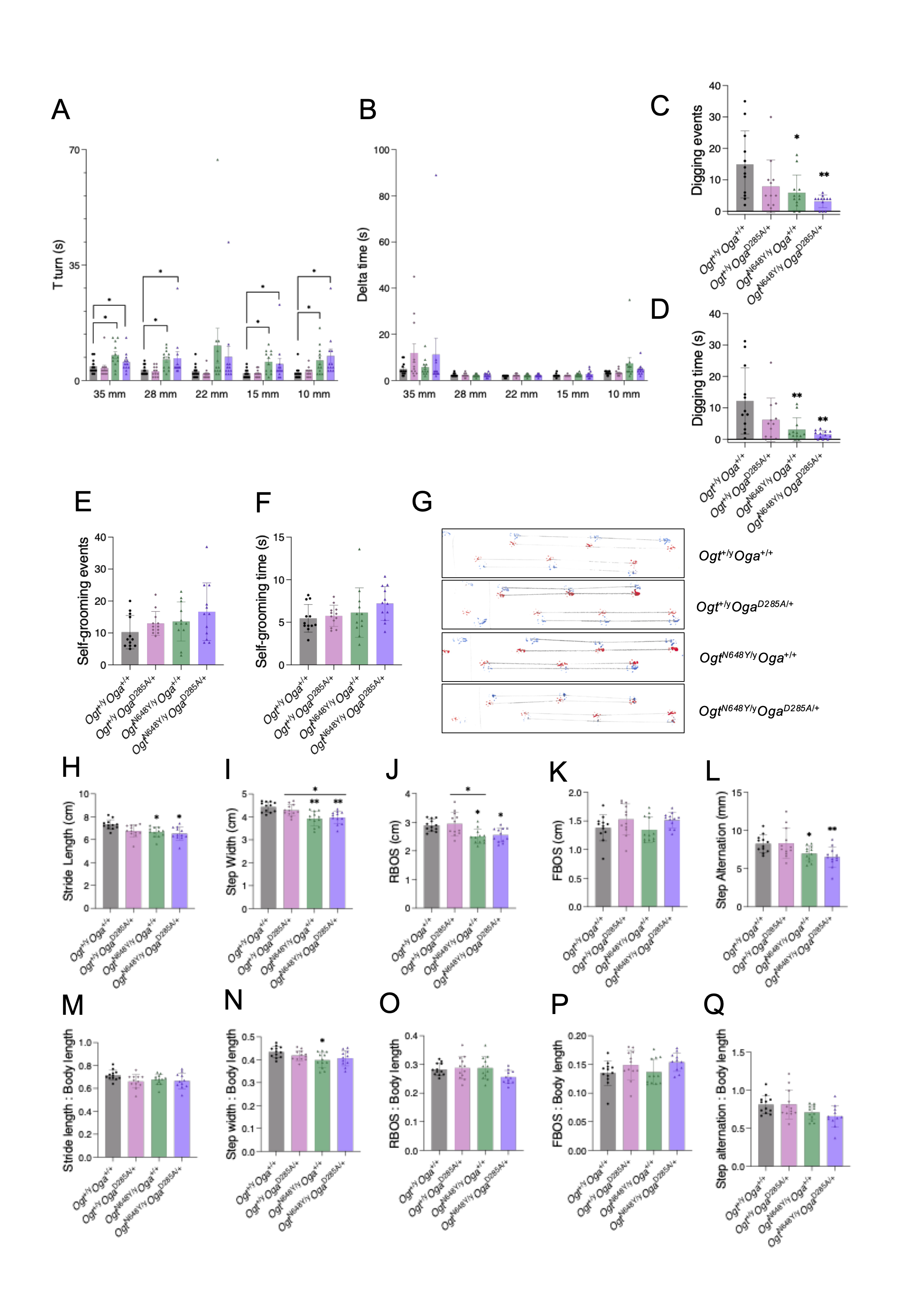

Supplement: Figure 3-2 — OgtN648Y/yOgaD285A/+ mice behaviour tests Data for behaviour tests performed in Ogt+/yOga+/+, Ogt+/yOgaD285A/+, OgtN648Y/yOga+/+ and OgtN648Y/yOgaD285A/+ mice. Data are represented as mean ± SD, n = 12 for all genotypes. One-way ANOVA (alpha, 0.05) is used for statistics. Significance shown as: *p < 0.05, **p < 0.01 and ***p < 0.001. (a) Time to perform the T-turn of Ogt+/yOga+/+ (black), Ogt+/yOgaD285A/+(pink), OgtN648Y/yOga+/+ (green) and OgtN648Y/yOgaD285A/+ (purple) mice during the static rods test. (b) Delta time (total time – time to perform the T-turn) of Ogt+/yOga+/+ (black), Ogt+/yOgaD285A/+(pink), OgtN648Y/yOga+/+ (green) and OgtN648Y/yOgaD285A/+ (purple) mice during the static rods test. (c) Number of digging events during 3 min observation. (d) Time spent digging during 3 min observation. (e) Number of self-grooming events during 3 min observation. (f) Time spent self-grooming during 3 min observation. (g) Representative footprints of Ogt+/yOga+/+, Ogt+/yOgaD285A/+, OgtN648Y/yOga+/+ and OgtN648Y/yOgaD285A/+ mice. Red footprints correspond to the forepaws; blue footprints correspond to the hind paws. Lines represent measured distances. (h) Measurement of stride length as the distance between footprints of the same hind paw. (i) Measurement of step width as the contralateral distance between hind and front paws. (j) Measurement of rear paws base of support (RBOS) as the contralateral distance between hind paws. (k) Measurement of front paws base of support (FBOS) as the contralateral distance between forepaws. (l) Measurement of step alternation as the ipsilateral distance between hind and front paws. (m) Stride length normalised to body length. (n) Step width normalised to body length. (o) RBOS normalised to body length. (p) FBOS normalised to body length. (q) Step alternation normalised to body length. Download Figure 3-2, TIF file. [file eneuro-13-ENEURO.0453-25.2026-s007.tif]

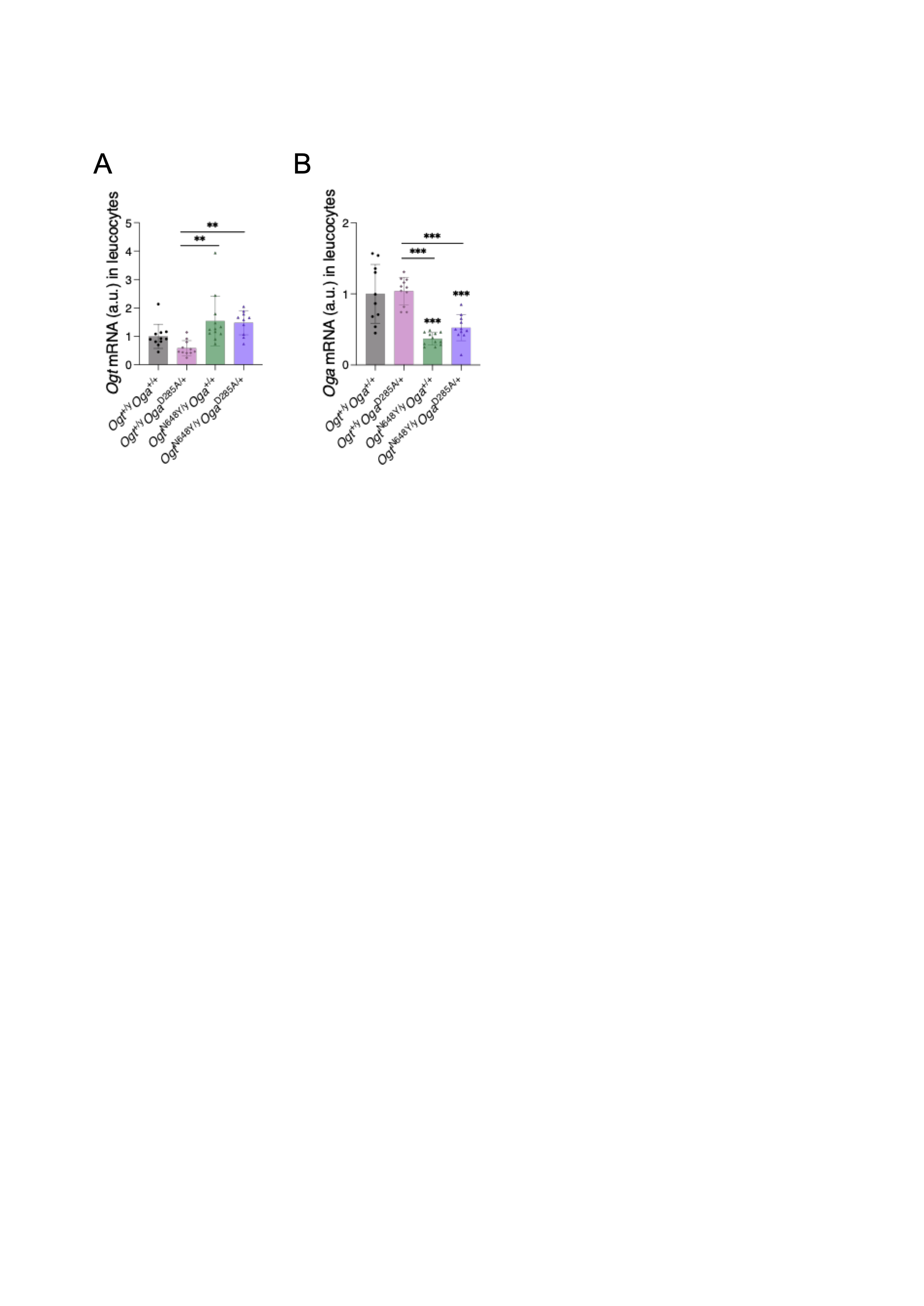

Supplement: Figure 3-3 — OgtN648Y/yOgaD285A/+ blood biochemistry Data for 20-week-old Ogt+/yOga+/+ (n = 11), Ogt+/yOgaD285A/+ (n = 11), OgtN648Y/yOga+/+ (n = 12), and OgtN648Y/yOgaD285A/+ (n = 11) mice. Data are represented as mean ± SD. One-way ANOVA (alpha, 0.05) is used for statistics. Significance shown as: *p < 0.05, **p < 0.01 and ***p < 0.001 (a) Quantification of Ogt mRNA levels in blood by RT-PCR. (b) Quantification of Oga mRNA levels in blood by RT-PCR. One Ogt+/yOga+/+ outlier was excluded from the analysis. Download Figure 3-3, TIF file. [file eneuro-13-ENEURO.0453-25.2026-s008.tif]
