# Supplementary material for: Genetic Rescue of Pathogenic O-GlcNAc Dyshomeostasis Associated with Microcephaly and Motor Deficits
Source: eNeuro. 2026 Jun 4;13(6):ENEURO.0453-25.2026. doi: 10.1523/ENEURO.0453-25.2026 (PMC13240978; doi:10.1523/ENEURO.0453-25.2026)
Supplement: Figure 2-2 — Table indicating absolute volumetry of 39 brain regions from T1 Mean and standard deviation of absolute volumes of the reported brain regions in Ogt+/y and OgtN684Y/y mice, and significance of p-values after multiple comparisons correction (alpha, 0.05). Download Figure 2-2, DOCX file. [file eneuro-13-ENEURO.0453-25.2026-s002.docx]

**Absolute volumetry of 39 brain regions from T1**

| **ROI** | **WT (Mean ± STD, mm³)** | **N648Y (Mean ± STD, mm³)** | **pVal (BHcorr)** |
| --- | --- | --- | --- |
| Amygdala | 14.20 ± 0.50 | 12.79 ± 0.52 | p<0.05 |
| Anterior Commissure | 1.95 ± 0.07 | 1.75 ± 0.06 | p<0.05 |
| Arbor Vita Of Cerebellum | 8.72 ± 0.30 | 7.86 ± 0.32 | p<0.05 |
| Basal Forebrain | 4.96 ± 0.18 | 4.47 ± 0.18 | p<0.05 |
| Bed Nucleus Of Stria Terminalis | 1.34 ± 0.05 | 1.22 ± 0.05 | p<0.05 |
| Cerebellar Cortex | 46.76 ± 1.66 | 42.10 ± 1.68 | p<0.05 |
| Cerebellar Peduncle | 2.79 ± 0.10 | 2.50 ± 0.10 | p<0.05 |
| Cerebral Cortex: Entorhinal Cortex | 10.47 ± 0.37 | 9.43 ± 0.38 | p<0.05 |
| Cerebral Cortex: Frontal Lobe | 42.67 ± 1.54 | 38.45 ± 1.57 | p<0.05 |
| Cerebral Cortex: Occipital Lobe | 6.57 ± 0.23 | 5.92 ± 0.24 | p<0.05 |
| Cerebral Cortex: Parieto-Temporal Lobe | 79.61 ± 2.87 | 71.66 ± 2.90 | p<0.05 |
| Cerebral Peduncle | 2.15 ± 0.08 | 1.94 ± 0.08 | p<0.05 |
| Colliculus: Inferior | 4.92 ± 0.17 | 4.45 ± 0.18 | p<0.05 |
| Colliculus: Superior | 8.16 ± 0.30 | 7.33 ± 0.31 | p<0.05 |
| Corpus Callosum | 17.31 ± 0.61 | 15.57 ± 0.62 | p<0.05 |
| Corticospinal Tract / Pyramids | 1.73 ± 0.06 | 1.55 ± 0.07 | p<0.05 |
| Dentate Gyrus Of Hippocampus | 3.79 ± 0.14 | 3.41 ± 0.14 | p<0.05 |
| Fimbria | 3.07 ± 0.11 | 2.72 ± 0.10 | p<0.05 |
| Globus Pallidus | 2.86 ± 0.10 | 2.57 ± 0.10 | p<0.05 |
| Hippocampus | 20.76 ± 0.73 | 18.68 ± 0.74 | p<0.05 |
| Hypothalamus | 10.93 ± 0.37 | 9.83 ± 0.40 | p<0.05 |
| Internal Capsule | 2.58 ± 0.09 | 2.33 ± 0.09 | p<0.05 |
| Lateral Olfactory Tract | 1.46 ± 0.05 | 1.32 ± 0.05 | p<0.05 |
| Lateral Septum | 3.46 ± 0.14 | 3.04 ± 0.10 | p<0.05 |
| Lateral Ventricle | 2.91 ± 0.11 | 2.62 ± 0.11 | p<0.05 |
| Medial Lemniscus / Medial Longitudinal Fasciculus | 2.09 ± 0.41 | 1.90 ± 0.33 | ns |
| Medial Septum | 1.52 ± 0.09 | 1.36 ± 0.06 | p<0.05 |
| Medulla | 25.62 ± 0.88 | 23.08 ± 0.93 | p<0.05 |
| Midbrain | 11.75 ± 0.42 | 10.56 ± 0.45 | p<0.05 |
| Nucleus Accumbens | 3.84 ± 0.14 | 3.46 ± 0.14 | p<0.05 |
| Olfactory Bulbs | 27.66 ± 0.95 | 25.37 ± 1.02 | p<0.05 |
| Olfactory Tubercle | 4.06 ± 0.15 | 3.65 ± 0.14 | p<0.05 |
| Optic Tract | 1.36 ± 0.05 | 1.23 ± 0.05 | p<0.05 |
| Periaqueductal Grey | 3.91 ± 0.16 | 3.50 ± 0.13 | p<0.05 |
| Pons | 15.34 ± 0.55 | 13.81 ± 0.54 | p<0.05 |
| Pre-Para Subiculum | 2.77 ± 0.10 | 2.49 ± 0.10 | p<0.05 |
| Striatum | 20.38 ± 0.72 | 18.35 ± 0.74 | p<0.05 |
| Thalamus | 16.24 ± 0.57 | 14.61 ± 0.59 | p<0.05 |
| Third Ventricle | 1.16 ± 0.09 | 1.04 ± 0.05 | p<0.05 |
