# Supplementary material for: Genetic Rescue of Pathogenic O-GlcNAc Dyshomeostasis Associated with Microcephaly and Motor Deficits
Source: eNeuro. 2026 Jun 4;13(6):ENEURO.0453-25.2026. doi: 10.1523/ENEURO.0453-25.2026 (PMC13240978; doi:10.1523/ENEURO.0453-25.2026)
Supplement: Figure 2-4 — Table indicating relative volumetry of 39 brain regions from T1 Mean and standard deviation of relative volumes of the reported brain regions in Ogt+/y and OgtN684Y/y mice, and significance of p-values after multiple comparisons correction (alpha, 0.05). Download Figure 2-4, DOCX file. [file eneuro-13-ENEURO.0453-25.2026-s004.docx]

**Relative volumetry of 39 brain regions from T1**

| **ROI** | **WT (Mean ± STD, %)** | **N648Y (Mean ± STD, %)** | **pVal (BHcorr)** |
| --- | --- | --- | --- |
| Amygdala | 3.14 ± 0.01 | 3.14 ± 0.00 | ns |
| Anterior Commissure | 0.43 ± 0.00 | 0.43 ± 0.00 | ns |
| Arbor Vita Of Cerebellum | 1.93 ± 0.01 | 1.93 ± 0.00 | ns |
| Basal Forebrain | 1.10 ± 0.00 | 1.10 ± 0.00 | ns |
| Bed Nucleus Of Stria Terminalis | 0.30 ± 0.00 | 0.30 ± 0.00 | ns |
| Cerebellar Cortex | 10.35 ± 0.03 | 10.35 ± 0.01 | ns |
| Cerebellar Peduncle | 0.62 ± 0.00 | 0.61 ± 0.01 | ns |
| Cerebral Cortex: Entorhinal Cortex | 2.32 ± 0.00 | 2.32 ± 0.00 | ns |
| Cerebral Cortex: Frontal Lobe | 9.45 ± 0.02 | 9.45 ± 0.01 | ns |
| Cerebral Cortex: Occipital Lobe | 1.45 ± 0.00 | 1.45 ± 0.00 | ns |
| Cerebral Cortex: Parieto-Temporal Lobe | 17.62 ± 0.04 | 17.61 ± 0.01 | ns |
| Cerebral Peduncle | 0.48 ± 0.00 | 0.48 ± 0.00 | ns |
| Colliculus: Inferior | 1.09 ± 0.01 | 1.09 ± 0.00 | ns |
| Colliculus: Superior | 1.81 ± 0.01 | 1.80 ± 0.01 | ns |
| Corpus Callosum | 3.83 ± 0.01 | 3.83 ± 0.01 | ns |
| Corticospinal Tract / Pyramids | 0.38 ± 0.01 | 0.38 ± 0.00 | ns |
| Dentate Gyrus Of Hippocampus | 0.84 ± 0.00 | 0.84 ± 0.00 | ns |
| Fimbria | 0.68 ± 0.01 | 0.67 ± 0.01 | ns |
| Globus Pallidus | 0.63 ± 0.00 | 0.63 ± 0.00 | ns |
| Hippocampus | 4.59 ± 0.01 | 4.59 ± 0.01 | ns |
| Hypothalamus | 2.42 ± 0.01 | 2.41 ± 0.02 | ns |
| Internal Capsule | 0.57 ± 0.00 | 0.57 ± 0.00 | ns |
| Lateral Olfactory Tract | 0.32 ± 0.00 | 0.32 ± 0.00 | ns |
| Lateral Septum | 0.77 ± 0.01 | 0.75 ± 0.02 | ns |
| Lateral Ventricle | 0.64 ± 0.00 | 0.64 ± 0.00 | ns |
| Medial Lemniscus / Medial Longitudinal Fasciculus | 0.46 ± 0.09 | 0.46 ± 0.07 | ns |
| Medial Septum | 0.34 ± 0.02 | 0.33 ± 0.00 | ns |
| Medulla | 5.67 ± 0.01 | 5.67 ± 0.01 | ns |
| Midbrain | 2.60 ± 0.01 | 2.60 ± 0.01 | ns |
| Nucleus Accumbens | 0.85 ± 0.00 | 0.85 ± 0.00 | ns |
| Olfactory Bulbs | 6.12 ± 0.11 | 6.23 ± 0.04 | ns |
| Olfactory Tubercle | 0.90 ± 0.00 | 0.90 ± 0.00 | ns |
| Optic Tract | 0.30 ± 0.00 | 0.30 ± 0.00 | ns |
| Periaqueductal Grey | 0.86 ± 0.01 | 0.86 ± 0.01 | ns |
| Pons | 3.40 ± 0.01 | 3.39 ± 0.01 | ns |
| Pre-Para Subiculum | 0.61 ± 0.00 | 0.61 ± 0.00 | ns |
| Striatum | 4.51 ± 0.01 | 4.51 ± 0.00 | ns |
| Thalamus | 3.59 ± 0.01 | 3.59 ± 0.01 | ns |
| Third Ventricle | 0.26 ± 0.02 | 0.26 ± 0.02 | ns |
